# Adverse events in women and children who have received intrapartum antibiotic prophylaxis treatment: a systematic review

**DOI:** 10.1186/s12884-017-1432-3

**Published:** 2017-07-26

**Authors:** Farah Seedat, Chris Stinton, Jacoby Patterson, Julia Geppert, Bee Tan, Esther R. Robinson, Noel Denis McCarthy, Olalekan A. Uthman, Karoline Freeman, Samantha Ann Johnson, Hannah Fraser, Colin Stewart Brown, Aileen Clarke, Sian Taylor-Phillips

**Affiliations:** 1 Division of Health Sciences, University of Warwick Medical School, Gibbet Hill Campus, Coventry, CV4 7AL UK; 2 Department of Obstetrics and Gynaecology, Birmingham Heartlands Hospital, Heart of England NHS Foundation Trust, Birmingham, B9 5SS UK; 3 Birmingham Public Health Laboratory (PHE), Heartlands Hospital, Birmingham, B9 5SS UK; 4 Bacteria Reference Department, National Infection Service, Public Health England, 61 Colindale Ave, London, NW95EQ UK

**Keywords:** *Streptococcus agalactiae*, Group B *Streptococcus*, Intrapartum antibiotic prophylaxis, Adverse events, Harms, Systematic review

## Abstract

**Background:**

Adverse events from intrapartum antibiotic prophylaxis (IAP) are poorly documented yet essential to inform clinical practice for neonatal group B *Streptococcus* (GBS) disease prevention. In this systematic review, we appraised and synthesised the evidence on the adverse events of IAP in the mother and/or her child.

**Methods:**

We searched MEDLINE, MEDLINE In-Process & Other Non-Indexed Citations, EMBASE, Cochrane, and Science Citation Index from date of inception until October 16th 2016. Reference lists of included studies and relevant systematic reviews were hand-searched. We included primary studies in English that reported any adverse events from intrapartum antibiotics for any prophylactic purpose compared to controls. The search was not restricted to prophylaxis for GBS but excluded women with symptoms of infection or undergoing caesarean section. Two reviewers assessed the methodological quality of studies, using the Cochrane Risk of Bias tool, and the Risk of Bias Assessment Tool for Nonrandomised Studies. Results were synthesised narratively and displayed in text and tables.

**Results:**

From 2364 unique records, 30 studies were included. Despite a wide range of adverse events reported in 17 observational studies and 13 randomised controlled trials (RCTs), the evidence was inconsistent and at high risk of bias. Only one RCT investigated the long-term effects of IAP reporting potentially serious outcomes such as cerebral palsy; however, it had limited applicability and unclear biological plausibility. Seven observational studies showed that IAP for maternal GBS colonisation alters the infant microbiome. However, study populations were not followed through to clinical outcomes, therefore clinical significance is unknown. There was also observational evidence for increased antimicrobial resistance, however studies were at high or unclear risk of bias.

**Conclusions:**

The evidence base to determine the frequency of adverse events from intrapartum antibiotic prophylaxis for neonatal GBS disease prevention is limited. As RCTs may not be possible, large, better quality, and longitudinal observational studies across countries with widespread IAP could fill this gap.

**Trial registration:**

CRD42016037195.

**Electronic supplementary material:**

The online version of this article (doi:10.1186/s12884-017-1432-3) contains supplementary material, which is available to authorized users.

## Background

Group B *Streptococcus* (GBS), or *Streptococcus agalactiae*, a gram-positive bacterium, is the leading cause of mortality and morbidity from neonatal sepsis [1]. GBS colonises the gastrointestinal and/or genitourinary tract in 10 to 30% of pregnant women [2–4], with a recent global rate of 17.9% [5]. If a pregnant woman is vaginally colonised with GBS when she is in labour, there is a 36% chance that GBS will be transmitted to her neonate [6]. Most GBS colonised neonates will be asymptomatic, however less than 1% may suffer from invasive early-onset GBS disease (less than seven days, EOGBS) [7]. Globally, culture-confirmed EOGBS has an estimated incidence of 0.43 per 1000 live births and a case fatality rate of 12.1%, which may be an underestimate [8].

To prevent EOGBS, the currently available prevention is intrapartum antibiotic prophylaxis (IAP), administered to mothers identified at risk of vertically transmitting GBS bacteria [9, 10]. The current recommendation for IAP in Western Europe, North America, and Australasia is intravenous penicillin (or ampicillin) given as soon as possible after the onset of labour and then every four hours until delivery, with intravenous cefazolin in the US, or clindamycin in the UK, for mothers allergic to penicillin [9–11]. Pregnant women are selected to be offered IAP using different policies. In some countries, women are offered IAP if they present with known risk factors for GBS, such as intrapartum fever or GBS bacteriuria, and in many other countries, women are actively screened for GBS colonisation at 35-37 weeks of pregnancy and treated in labour if they are positive [12]. Screening for GBS maternal colonisation is controversial as up to 30% of women with positive results at 35-37 weeks revert to negative by labour [13], and the large majority of women who are colonised with GBS during labour have healthy neonates who will not suffer from EOGBS.

A number of potential harms have been suggested as a result of the widespread use of IAP associated with neonatal GBS prevention [14, 15, 10]. IAP has been associated with antimicrobial resistance [16–18], neonatal infections caused by gram-negative bacteria [19, 15, 18], *Clostridium difficile* infection in mothers [20], maternal anaphylaxis, which although very rare, can be fatal for mother and baby [9], neonatal microbiota changes that could lead to short and long-term health problems [21–23], anxiety for the mother, family, and medical staff, and the medicalisation of labour [15, 10].

The potential harms from IAP are poorly documented and understood, and there has been no systematic review of the evidence. This information is essential to assess whether the benefits of IAP treatment for neonatal GBS disease prevention outweigh the harms. Therefore, we conducted a systematic review to identify, appraise, and synthesise the evidence on the adverse events experienced by the mother and/or her child after receiving IAP treatment. This review was conducted as part of a national review on whether the UK should introduce a GBS screening programme, a crucial part of these screening reviews is to understand the harms of treatment.

## Methods

This systematic review is reported according to PRISMA guidelines [24]. As this was a secondary analysis of existing data, ethical consent was not required.

### Search strategy

Searches were conducted in MEDLINE, MEDLINE In-Process & Other Non-Indexed Citations, EMBASE, Cochrane Library: Cochrane Database of Systematic Reviews, CENTRAL, DARE and HTA databases, and Science Citation Index Expanded from date of inception until October 16th 2016. The search strategy combined both text words and MeSH terms for antibiotic prophylaxis, labour, and adverse events, and was limited to English and humans (see Additional file 1 for complete search strategy). We used recommended search filters for adverse events [25, 26], and systematically included terms for known IAP adverse events from previous studies [14, 15, 27, 10] and expert opinion. We also hand-searched reference lists of included studies and relevant systematic reviews, and experts cross-checked included studies.

### Eligibility criteria and study selection

Two reviewers independently screened the titles, abstracts, and full texts of all identified records. Disagreements were resolved by discussion, with involvement of a third reviewer if necessary. We included any full text randomised controlled trials (RCTs), cohort studies, or case-control studies in English, reporting any adverse events experienced by mothers and/or their children after being exposed to antibiotics during labour for any prophylactic purpose, compared to an unexposed control group. As the evidence base on IAP for neonatal GBS disease prevention is limited, we included studies on IAP for any prophylactic indication. IAP studies for caesarean sections or symptomatic mothers, those in which women were given antibiotics before labour, or neonates given antibiotics after birth were excluded. Studies were included if 90% or more of the study population met the inclusion criteria, or if results for those who met the inclusion criteria were reported separately. We excluded case series, case reports, abstracts, editorials, letters, books, consensus statements, opinions, and reviews.

### Quality appraisal

Two reviewers independently appraised the risk of bias for each included study using the Cochrane Risk of Bias (RoB) tool [28], and the Risk of Bias Assessment Tool for Nonrandomised Studies (RoBANS) [29]. Selection, performance, detection, attrition, and reporting biases were assessed and classified as low, high, and unclear risk of bias.

### Data extraction and synthesis

Meta-analyses could not be performed due to the heterogeneity across the adverse outcomes assessed. Narrative syntheses were conducted, and the results of individual studies displayed in text and tables. Missing statistical parameters of importance were calculated if data permitted. Odds ratios (ORs) were calculated for case-control studies and risk ratios (RRs) and risk differences (RDs) were calculated for all other study designs using Stata version 13 (Stata Corp, College Station, Texas).

## Results

### Characterisation of included studies

**Fig. 1:**
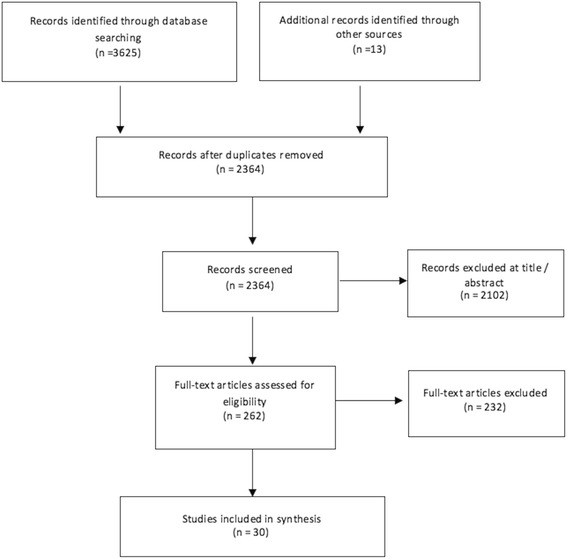
Flow diagram of study selection

Our search identified 2364 unique references. After sifting titles and abstracts, 262 full texts were screened, of which 30 studies met the inclusion criteria and were included in the synthesis (see Fig. 1 for study flow and Additional file 1 for full text studies excluded with reason) [30–59]. Fourteen were cohort studies [30, 32, 33, 46, 35–37, 39, 42, 44, 45, 56, 31, 57], three case-control [34, 38, 54], 12 RCTs [40, 47–53, 55, 43, 41, 58], and one a sub-study [59] of an included RCT [58]. Nine studies investigated IAP for GBS prevention [30, 34, 46, 35, 36, 39, 54, 31, 57], two for GBS prevention and other indications [37, 42], three for post-partum infection prevention [40, 49, 50], eight for preterm labour [41, 43, 47, 48, 51–53, 55], two for neonatal sepsis prevention [58, 59], and six did not state the indication (see Additional file 1: Table S1 for study characteristics) [32, 33, 38, 45, 44, 56]. Some IAP effectiveness trials reported outcomes, such as neonatal and maternal infection, that could plausibly increase from IAP due to changes in the organisms causing infections and/or antibiotic susceptibility [18, 44], but could also decrease if the IAP is successful [40, 43, 47–53, 55, 58]. To prevent bias in reporting, we reported these outcomes irrespective of whether they were identified as benefits or harms (see bottom of Additional file 1: Table S1).

### Methodological quality

**Fig. 2:**
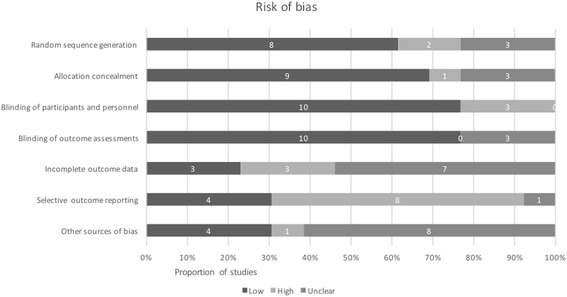
Risk of bias in randomised controlled trials according to the Cochrane RoB [28]

None of the RCTs were judged as low risk of bias across all domains as assessed by the Cochrane Risk of Bias tool (see Fig. 2) [28]. The greatest risk of bias was in selective outcome reporting, where eight RCTs were at high risk partly or solely because the definition and measurement of side effects was not pre-specified in the methods but only reported in the results [47–50, 53, 55, 40, 59]. More than half of the RCTs were rated as having unclear risk of bias for incomplete outcome data as there was substantial missing data, for example, on adverse events in the control group [40, 49–51, 53, 58, 55]. We noted a number of other sources of bias across RCTs, including relatively small sample sizes [41, 52, 59], data not presented [41, 49, 50], a lack of information on treatment regimens [48] and details of intention to treat analysis [51, 55], inaccuracies in the numbers provided for participant flow [53], and parent-reported outcomes rather than objective assessment [41].

**Fig. 3:**
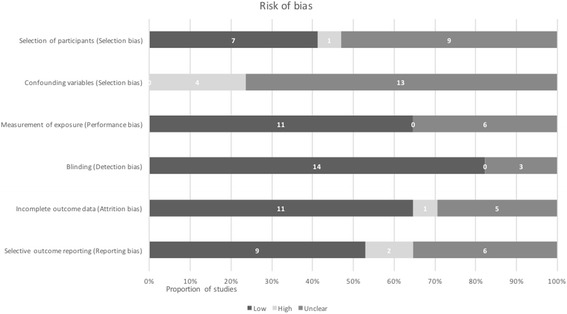
Risk of bias in non-randomised studies according to RoBANS [29]

There were no observational studies judged as low risk of bias across all domains on the RoBANS tool (see Fig. 3) [29]. The confounding variables domain had the highest concern, as four studies were rated as high risk [30, 37, 44, 46], none as low risk, and 13 as unclear risk of bias [32–36, 38, 39, 42, 45, 54, 56, 31, 57], as some variables were accounted for in the study design or at least reported, while others, such as maternal risk factors, prenatal antibiotics, and caesarean sections, were not. Likewise, selection of participants was also unclear across nine studies [32, 33, 39, 44, 45, 54, 56, 31, 57], as there was no mention of how participants were selected and/or some important baseline characteristics were not reported.

### Adverse events associated with IAP

A range of child and maternal adverse events were investigated for association with IAP including maternal thrush, childhood atopic dermatitis, neonatal infections and respiratory distress, necrotising enterocolitis, and *Clostridium* difficile bowel problems (see Additional file 1: Table S1 for summary of results). Below we present the findings on three key results – gut microbiota, antibiotic resistance, and long-term adverse events.

#### Gut microbiota

Seven cohort studies consistently showed that IAP alters the infant microbiome [30, 32, 33, 36, 39, 31, 57]. At day 2, 3, 6-7, 10, 30, and 90, there were differences in the relative composition and the colony forming units per gram (log CFU/g) of organisms in the gut of infants whose mothers were and were not treated with IAP (see Tables 1 and 2 for results) [32, 33, 39, 31, 57, 36]. Two studies also reported on sample richness and biodiversity, finding that at day 6-7 and day 30, infants whose mothers were treated with IAP had a less diverse microbial profile compared to controls [31, 57]. At day 6-7, there was also a clear segregation between the microbiota profiles of IAP compared to control infants when they were plotted on principal coordinate analysis plots, which disappeared by day 30 [31, 57]. Similar to gut microbiota, Keski-Nisula et al. (2013), [42] found a decreased transmission of vaginal *Lactobacillus*-dominant mixed flora on oral surfaces in neonates whose mothers were treated with IAP compared to those who were not (1 versus 13, OR = 0.08 95% CI 0.007–0.80). While there was consistent evidence on gut microbiota alterations, it is unclear if any of the alterations are related to clinical adverse events or not.

**Table 1:** Qualitative gut microbiota composition of IAP-treated and untreated infants

Organism	Study	Number of infants (*n*) in each group	Relative abundance in microbiota composition (%) or number of infants (*n*) colonised					
			Day 1	Day 2	Day 3	Day 6/ 7	Day 10	Day 30
Phyla								
All	Arboleya 2016 [33]	IAP *n* = 14 Control *n* = 13	No differences					
*Actinobacteria*	Aloisio 2016 [31]^a^	IAP *n* = 10 Control *n* = 10				IAP: 0.4% Control: 3.8% *p* < 0.05		
	Arboleya 2016 [33]	IAP *n* = 14 Control *n* = 13						Lower % in IAP, *p* < 0.05
	Mazzola 2016 [57]^a^	Breast-fed IAP *n* = 7 Breast-fed Control *n* = 7				IAP: 0% Control: 17% *p* < 0.001		
		Mixed-fed IAP *n* = 6 Mixed-fed Control *n* = 6				IAP: 1% Control: 8% *RR 0.13 (CI 0.02-0.98)*		IAP: 7%
*Bacteriodetes*	Aloisio 2016 [31]^a^	IAP *n* = 10 Control *n* = 10				IAP: 16% Control: 47.7% *p* < 0.05		
	Mazzola 2016 [57]^a^	Mixed-fed IAP *n* = 6 Mixed-fed Control *n* = 6				IAP: 21% Control: 36% *RR 0.59 (CI 0.3 –0.93)*		IAP: 34% Control: 26% *RR 1.31 (*CI *0.85-2.01)*
*Proteobacteria*	Aloisio 2016 [31]^a^	IAP *n* = 10 Control *n* = 10				IAP: 54.7% Control: 15.5% *p* < 0.05		
	Arboleya 2016 [33]	IAP *n* = 14 Control *n* = 13						Higher % in IAP, *p* < 0.001
	Mazzola 2016 [57]^a^	Breast-fed IAP *n* = 7 Breast-fed Control *n* = 7				Higher % in IAP, *p* < 0.062		
		Mixed-fed IAP *n* = 6 Mixed-fed Control *n* = 6				IAP: 37% Control: 17% *RR 2.18 (CI 1.32-3.60)*		IAP: 28%
*Firmicutes*	Arboleya 2016 [33]	IAP *n* = 14 Control *n* = 13						Lower % in IAP, *p* < 0.01
	Mazzola 2016 [57]^a^	Mixed-fed IAP *n* = 6 Mixed-fed Control *n* = 6				IAP: 41% Control: 29% *RR 1.14 (CI 0.96-2.08)*		IAP: 30%
Family								
*Bifidobacteriaceae*	Aloisio 2016 [31]^a^	IAP *n* = 10 Control *n* = 10				IAP: 0.02% Control: 6.47% *p* < 0.05		
	Arboleya 2015 [32]	IAP *n* = 14 Control *n* = 13						Lower % in IAP, *p* < 0.05
*Comamonadaceae*	Arboleya 2015 [32]	IAP *n* = 14 Control *n* = 13						Lower % in IAP, *p* < 0.05
*Enterobacteriaceae*	Arboleya 2015 [32]	IAP *n* = 14 Control *n* = 13						Higher % in IAP, *p* < 0.05
	Mazzola 2016 [57]^a^	Breast-fed IAP *n* = 7 Breast-fed Control *n* = 7				Higher % in IAP, *p* = 0.044		IAP: 44% Control: 16% *RR 2.75 (CI 1.67-4.54)*
		Mixed-fed IAP *n* = 6 Mixed-fed Control *n* = 6				IAP: 35% Control: 17% *RR 2.06 (CI 1.24-3.42)*		IAP: 28%
	Jaureguy 2004 [39]^a^	IAP *n* = 25 Control *n* = 25			IAP *n* = 13 Control *n* = 16 *p* = 0.58	IAP: 0%		
*Lachnospiraceae*	Mazzola 2016 [57]^a^	IAP *n* = 14 Control *n* = 13						IAP: 4%
*Leuconostaceae*	Arboleya 2015 [32]	IAP *n* = 14 Control *n* = 13		Lower % in IAP, *p* < 0.05				
*Micrococcaceae*	Arboleya 2015 [32]	IAP *n* = 14 Control *n* = 13					Lower % in IAP, *p* < 0.05	
*Propionibacteriaceae*	Arboleya 2015 [32]	IAP *n* = 14 Control *n* = 13					Lower % in IAP, *p* < 0.05	
*Staphylococcaceae*	Arboleya 2015 [32]	IAP *n* = 14 Control *n* = 13						Lower % in IAP, *p* < 0.05
*Streptococcaceae*	Arboleya 2015 [32]	IAP *n* = 14 Control *n* = 13						Lower % in IAP, *p* < 0.05
*Veillonellaceae*	Mazzola 2016 [57]^a^	Breast-fed IAP *n* = 7 Breast-fed Control *n* = 7						Lower % in IAP, *p* = 0.035
Unclassified *Actinobacteria*	Arboleya 2015 [32]	IAP *n* = 14 Control *n* = 13						Lower % in IAP, *p* < 0.05
Unclassified *Bacilli*		IAP *n* = 14 Control *n* = 13						Lower % in IAP, *p* < 0.05
Unclassified *Lactobacillales*		IAP *n* = 14 Control *n* = 13						Lower % in IAP, *p* < 0.05
Genera								
*Bacteroides*	Jaureguy 2004 [39]^a^	IAP *n* = 25 Control *n* = 25			IAP *n* = 13 Control *n* = 7 *p* = 0.15			
	Mazzola 2016 [57]^a^	Breast-fed IAP *n* = 7 Breast-fed Control *n* = 7				IAP: 7% Control: 20% *p* = 0.078		
		Mixed-fed IAP *n* = 6 Mixed-fed Control *n* = 6				IAP: 13% Control: 32% *RR 0.41 (CI 0.23-0.73)*		
*Bifidobacteria*	Jaureguy 2004 [39]^a^	IAP *n* = 25 Control *n* = 25			IAP *n* = 6 Control *n* = 12 *p* = 0.18			
	Mazzola 2016 [57]^a^	Breast-fed IAP *n* = 7 Breast-fed Control *n* = 7				IAP: 0% Control: 16% *p* = 0.001		IAP: 6% (compared to day 7, *p* = 0.025) Control: 6%
	Mazzola 2016 [57]^a^	Mixed-fed IAP *n* = 6 Mixed-fed Control *n* = 6				IAP: 1% or 0% Control: 5%		IAP: 6% (compared to day 7, *p* = 0.013) Control: 19% *RR: 0.32 (CI 0.13-0.76)*
*Clostridia*	Jaureguy 2004 [39]^a^	IAP *n* = 25 Control *n* = 25			IAP *n* = 3 Control *n* = 10 *p* = 0.04			
*Enterococci*	Jaureguy 2004 [39]^a^	IAP *n* = 25 Control *n* = 25			IAP *n* = 15 Control *n* = 17 *p* = 0.73			
*Escherichia*	Mazzola 2016 [57]^a^	Breast-fed IAP *n* = 7 Breast-fed Control *n* = 7				IAP: 52% Control: 14% *RR 3.71 (CI 2.21-6.25)*		
*Staphylococci*	Jaureguy 2004 [39]^a^	IAP *n* = 25 Control *n* = 25			IAP *n* = 21 Control *n* = 22 *p* = 1.00			
*Streptococci*	Mazzola 2016 [57]^a^	Mixed-fed IAP *n* = 6 Mixed-fed Control *n* = 6				IAP: 32% Control: 10% *RR 3.2 (CI 1.66-6.15)*		IAP: 8% (compared to day 7, *p* = 0.042)
Other microbial genus	Aloisio 2016 [31]^a^	IAP *n* = 10 Control *n* = 10				No significant differences		

CI confidence interval, IAP intrapartum antibiotic prophylaxis, *p* probability value, RR risk ratio

*Numbers in italics calculated by reviewers*

^a^Group B *Streptococcus* prophylaxis

**Table 2:** Quantitative gut microbiota composition in IAP-treated and untreated infants

Organism	Study	Number of infants (*n*) in each group	Log colony forming units per gram (CFU/g)			
			Day 3	Day 6/7	Day 30	Day 90
Family						
*Staphylococcaceae*	Arboleya 2015 [32]	IAP *n* = 14 Control *n* = 13			Lower log cells/g in IAP, *p* < 0.05	
*Enterobacteriaceae*	Arboleya 2015 [32]	IAP *n* = 14 Control *n* = 13			Higher log cells/g in IAP, *p* < 0.05	
	Jaureguy 2004 [39]^a^	IAP *n* = 25 Control *n* = 25	IAP: Md 8.4 (R 3.3-9.5) Control: Md 9.2 (R 3.3-9.8) *p* = 0.18			
Genera						
*Bacteroides*	Jaureguy 2004 [39]^a^	IAP *n* = 25 Control *n* = 25	IAP: Md 8.0 (R 6.3-10.3) Control: Md 7.9 (R 3.6-9.6) *p* = 0.12			
*Bifidobacteria*	Jaureguy 2004 [39]^a^	IAP *n* = 25 Control *n* = 25	IAP: Md 8.2 (R 4.3-9.5) Control: Md 8.5 (R 6.9-10.3) *p* = 0.10			
	Arboleya 2015 [32]	IAP *n* = 14 Control *n* = 13				Lower log cells/g in IAP, *p* < 0.05
*Bifidobacterium spp.*	Aloisio 2014 [30]^a^	IAP *n* = 26 Control *n* = 26		IAP: M 5.85 (R 3.24-7.79) Control: M 7.29 (R 4.12-10.95) *p* = 0.001		
	Corvaglia 2016 [36]^a^	IAP *n* = 35 Control *n* = 29		IAP: Md 6.01 (IQR 5.51-6.98) Control: Md 7.80 (IQR 6.61-8.26) *p* = 0.000	IAP: Md 8.41 (IQR 7.71-8.80) Control: Md 8.39 (IQR 7.96-8.86) *p* = 0.363	
	Mazzola 2016 [57]^a^	Breast-fed IAP *n* = 7 Breast-fed Control *n* = 7		IAP: Md 5.86 Control: Md 8.16 *p* = 0.005	IAP: Md 7.72 (compared to day 7, *p* = 0.035) Control: Md 8.62 NS	
		Mixed-fed IAP *n* = 6 Mixed-fed Control *n* = 6		IAP: Md 5.81 Control: Md 7.19 *p* = 0.03	IAP: Md 8.50 (compared to day 7, *p* = 0.036) Control Md 8.55 (compared to day 7, *p* = 0.028)	
*Clostridia*	Jaureguy 2004 [39]^a^	IAP *n* = 25 Control *n* = 25	IAP: Md 5.3 (R 4.3-5.8) Control: Md 6.2 (R 3.6-8.1) *p* = 0.01			
*Enterococci*	Jaureguy 2004 [39]^a^	IAP *n* = 25 Control *n =* 25	IAP: Md 8.3 (R 3.6-10.3) Control: Md 7.3 (R 3.3-9.5) *p* = 0.78			
*Lactobacillus spp*.	Aloisio 2014 [30]^a^	IAP *n* = 26 Control *n* = 26		IAP: M 6.69 (R 5.40-8.93) Control: M 6.73 (R 5.45-8.20) NS		
	Corvaglia 2016 [36]^a^	IAP *n* = 35 Control *n* = 29		IAP: Md 5.56 (IQR 4.94-6.14) Control: Md 5.45 (IQR 4.81-6.14) *p* = 0.872	IAP: Md 5.29 (IQR 4.68–6.01) Control: Md 5.25 (IQR 4.60-6.15) *p* = 0.932	
*Staphylococci*	Jaureguy 2004 [39]^a^	IAP *n* = 25 Control *n* = 25	IAP: Md 6.5 (R 3.6-8.0) Control: Md 7.0 (R 4.0-9.3) *p* = 0.53			
Species						
*Escherichia coli*	Aloisio 2014 [30]^a^	IAP *n* = 26 Control *n* = 26		IAP: M 8.18 (R 4.09-12.70) Control: M 9.03 (R 5.61-11.78) NS		
*Bacteroides fragilis*	Aloisio 2014 [30]^a^	IAP *n* = 26 Control *n* = 26		IAP: M 8.17 (R 4.68-11.99) Control: M 8.53 (R 5.22-11.16) NS		
	Corvaglia 2016 [36]^a^	IAP *n* = 35 Control *n* = 29		IAP: Md 7.71 (IQR 5.80-9.33) Control: Md 7.75 (IQR 5.87-9.61) *p* > 0.05	IAP: Md 7.36 (IQR 5.80-9.09) Control: Md 8.51 (IQR 5.86-9.37) *p* > 0.05	
*Clostridium difficile*	Aloisio 2014 [30]^a^	IAP *n* = 26 Control *n* = 26		IAP: M 3.89 (R 3.12-4.80) Control: M 3.70 (R 2.85-5.46) NS		
*Total bacteria*	Mazzola 2016 [57]^a^	Breast-fed IAP *n* = 7 Breast-fed Control *n* = 7 Mixed-fed IAP *n* = 6 Mixed-fed Control *n* = 6		All groups: R 9.38-9.71	All groups: R 9.53-9.83	
	Arboleya 2015 [32]	IAP *n* = 14 Control *n* = 13			Higher log cells/g in IAP, *p* < 0.05	

IAP intrapartum antibiotic prophylaxis, M mean, Md median, R range, IQR interquartile range, *p* probability value

^a^GBS prophylaxis

#### Antimicrobial resistance

Six studies reported antimicrobial resistance. Of the RCTs, Gordon et al. (1995) reported zero cases of multi-resistant bacterial infections in the intervention group of 58 infants whose mothers were treated with IAP for preterm labour [48]. Roca et al. (2016) investigated GBS, *Staphylococcus aureus* (*S. aureus*), and *Streptococcus pneumoniae* (*S. pneumoniae*) resistant to azithromycin in 829 mothers and 843 infants treated with azithromycin for neonatal sepsis prevention [58]. *S. aureus* resistant to azithromycin were found at day 3 in maternal breast milk, at day 6 in newborn nasopharynx and maternal breast milk, at day 8 in vaginal swabs, and at day 14 and day 28 in newborn and maternal nasopharynx and maternal breast milk. *S. pneumoniae* resistant to azithromycin were also identified in the maternal nasopharynx, which occurred at day 28 only (see Additional file 1: Table S1).

Of the four observational studies, Glasgow et al. (2005) found that in 62 infants whose mothers were treated with various IAP drugs (indication not stated), 24 (39%) had ampicillin-resistant organisms, compared to 13/120 (11%) infants whose mothers were not treated (OR = 5.7 95% CI 2.3-14.3) [38]. The authors also reported a significant difference when analysing ampicillin-resistant bacteria causing urinary tract infections separately (OR = 4.3 95% CI 1.6-11.7). Similarly, Stoll et al. (2002) found that mothers of infants with ampicillin-resistant strains of *Escherichia coli* (*E. coli*) were more likely to have received intrapartum ampicillin than those with ampicillin-sensitive strains (26 of 28 [93%] versus 1 of 5 [20%] *p* = 0.01) [44]. It was unclear whether the infants in these two studies were treated with antibiotics before they were tested for antimicrobial resistance. Ashkenazi-Hoffnung et al. (2011) did not find any differences between 17 infants born to mothers treated with IAP for GBS prevention and 178 infants who were not, in first generation cephalosporin resistance in *E. coli* (60% versus 22.7% *p* = 0.21) or any bacteria causing late-onset serious bacterial infections (57% versus 26% *p* = 0.19), or ampicillin resistance in *E. coli* (100% versus 54.5% *p* = 0.14) or any bacteria causing late-onset serious bacterial infections (85% versus 63% *p* = 0.19) [34]. The authors did find higher development of first generation cephalosporin-resistant urinary tract infections (75% versus 23.5% *p* = 0.04). Lastly, Jaureguy et al. (2004) did not find a difference in the number of infants colonised with amoxicillin-resistant *Enterobacteriaceae* (10/25 [40%] versus 12/25 [48%], calculated RR = 0.83 95% CI 0.44-1.56) and amoxicillin-resistant *E. coli* (6/25 [24%] versus 11/25 [44%] calculated RR = 0.55 95% CI 0.24-1.25) in the gut of infants whose mothers were or were not treated with IAP [39].

#### Long-term adverse events

Kenyon et al. (2008) was the only RCT that reported on the long-term adverse events of IAP [41]. They found that IAP may be associated with severe consequences of functional impairment and cerebral palsy, as well as bowel problems in a factorial randomised trial comparing children aged seven whose mothers had received any erythromycin (erythromycin alone or combined with amoxicillin-clavulanate) compared to no erythromycin, and any amoxicillin-clavulanate (alone or with erythromycin) compared to no amoxicillin-clavulanate for preterm labour. The risk of cerebral palsy was higher in infants whose mothers received any erythromycin versus no erythromycin (placebo or amoxicillin-clavulanate) (53/1611 [3%] and 27/1562 [2%] OR = 1.93 95% CI 1.21-3.09) or any amoxicillin-clavulanate versus no amoxicillin-clavulanate (placebo or erythromycin) (50/1587 [3%] and 30/1586 [2%] OR = 1.69 95% CI 1.07–2.67). More children who developed cerebral palsy had been born to mothers who had received both antibiotics (35/735) than to mothers who received erythromycin only (18/785), amoxicillin-clavulanate only (15/763), or double placebo (12/735) (both drugs versus double placebo: OR = 2.91 95% CI 1.50–5.65). The authors also found that any erythromycin significantly increased the risk of bowel problems (64/1611 [4%] versus 38/1562 [2%] OR = 1.66 95% CI 1.10-2.49) and functional impairment (658/1554 [42%] versus 574/1498 [38%] OR = 1.18 95% CI 1.02–1.37) compared to no erythromycin. None of these effects were found for either erythromycin or amoxicillin-clavulanate alone compared to placebo, however this may have been a result of insufficient power.

The study had a low risk of bias in all major domains, however there were critical limitations. Multiple statistical comparisons were conducted on a relatively small sample size increasing the probability of getting a significant effect due to chance. In addition to cerebral palsy, functional impairment, and bowel problems, the authors also investigated diabetes, behavioural problems, educational attainment, attention deficit hyperactivity disorder, and other developmental problems, and did not find any significant differences between any of the treatment and control groups. This is particularly important as the biological plausibility of IAP increasing the risk of cerebral palsy is unknown.

## Discussion

In this systematic review, we presented the evidence on adverse events experienced by the mother and/or her child after treatment with intrapartum antibiotic prophylaxis (IAP). Despite a wide range of adverse outcomes reported from 17 observational studies and 13 RCTs, there was limited high quality information to determine the frequency of adverse events from IAP for neonatal GBS disease prevention. The evidence contains much uncertainty, with a substantial evidence gap around the long-term effects of IAP. The only RCT investigating the long-term effects of IAP reported a moderate effect of severe consequences such as cerebral palsy. This trial had limited applicability as it used a different drug, a longer drug regimen, and pre-term rather than term labour compared to IAP for GBS prevention. We also found consistent observational evidence that IAP for neonatal GBS prevention alters the infant microbiome, with some studies showing changes up to 90 days of life. However, these study populations were not followed through to clinical outcomes, therefore the short- and long-term clinical significance of the changes are unknown. Finally, there was evidence for increased antibiotic resistance in some, but not all studies, with no evidence of a reduction. However, this observational evidence was at high or unclear risk of bias due to confounding variables.

Our review is the first systematic assessment of the literature on the adverse events from IAP. We had an extensive search with no date limit, and referencing checking of all included papers and relevant systematic reviews. We also had expert input, two reviewers conducting processes, including quality appraisal using validated tools, and we calculated summary measures for study outcomes where they were not available. However, as our search was broad and focused heavily on harms or adverse events search terms, we may not have found studies which investigated an outcome that would potentially be an adverse event, but may not have been indexed as such. Furthermore, as we only included studies for which full texts were available in English, adverse events reported in other languages may have been missed. We were also unable to conduct any meta-analyses due to the heterogeneity across the adverse events investigated.

Previous literature has suggested that initial bacterial colonisation of the gut plays an important role in the development of the mucosal immune system of infants [21–23, 42]. Microbiota changes from antibiotics have been associated with respiratory problems in children such as asthma, metabolic problems such as obesity and diabetes, and autism [21–23]. For example, Cox et al. (2014) demonstrated that low dose penicillin delivered after birth caused gut microbiota changes that led to permanent abnormalities in metabolism and immunity in mice [60]. More recently, antibiotic exposure before six months of age or repeatedly during infancy was associated with increased body mass and height in healthy children [23]. The antibiotics in these studies were not administered intrapartum. Long-term follow-up investigations linking antibiotic prophylaxis specifically during labour and early microbiota alterations to clinical consequences are required to understand their significance.

Evidence on the increase of antibiotic resistance after IAP was inconsistent. Literature on the trends of antibiotic resistance in countries offering IAP for neonatal GBS prevention has shown an increase in rates overtime. Resistance to clindamycin and erythromycin has increased in the last 20 years [9], with reported resistance to erythromycin at 30% or higher in the US and Switzerland, and above 15% for clindamycin in the US, Switzerland, and England [61–63]. Similarly, although GBS remains almost universally susceptible to penicillin [16], in 2005 in the US, 0.2% of GBS isolates had reached the upper level of susceptibility [61, 64]. However, these trends are difficult to attribute specifically to IAP for GBS prevention, and could be due to other factors.

Kenyon et al.’s (2008) RCT had a low risk of bias and showed that IAP was associated with an increase in the severe consequences such as cerebral palsy [41]. However, the effect size was small, and with multiple statistical comparisons conducted on the same population, the probability of a chance result is increased. Based on previous literature the plausible biological mechanisms through which IAP may cause the development of cerebral palsy are unknown [41, 65]. Complicating these findings, a second trial on IAP for pregnant women with preterm rupture of the membranes (excluded due to signs of infection confounding IAP effects) found no difference in the proportion of children with cerebral palsy between treated or untreated women [66]. Therefore, why cerebral palsy occurred in the first study and whether it would occur as a result of use of IAP for neonatal GBS prevention, which involves different drug regimens and durations, is uncertain.

A Cochrane meta-analysis concluded that despite an 83% reduction in EOGBS incidence from IAP, IAP for maternal GBS is not supported by conclusive evidence due to a high risk of bias across RCTs [67]. Combining this uncertainty with the results of this review makes it increasingly difficult to ascertain whether the benefits of administering IAP for EOGBS prevention outweigh the harms to mothers and children. Large, well-designed RCTs required to answer this question may no longer be feasible as IAP is now the recommended treatment. Instead, large, better quality, and longitudinal observational studies across countries with widespread IAP may be an alternative to understand the adverse events occurring in participants treated with IAP. Expanding EOGBS prevention from risk-based strategies to universal antenatal screening introduces the risk of increasing the number of low risk women treated with IAP. Up to 30% of mothers positive in pregnancy may become negative by birth, and less than 1% of mothers colonised in labour have a baby with EOGBS, all of whom could be unnecessarily exposed to potential harms [13, 7]. As observational evidence on universal GBS screening effectiveness is limited due to inherent biases [68, 9], an RCT could inform on both the effectiveness and harms of screening and IAP treatment for neonatal GBS disease prevention.

## Conclusions

The evidence on the adverse events from IAP treatment for neonatal GBS disease prevention is unclear, inconsistent, and/or at risk of bias. There is consistent evidence that GBS antibiotic prophylaxis alters the infant microbiome, and some inconsistent evidence that IAP increases antibiotic resistance. However, this evidence is at risk of bias, and the clinical consequences of the microbiome alterations are unknown. There is evidence from a single long-term RCT associating IAP in pre-term labour with potentially severe consequences such as cerebral palsy, however, it has applicability concerns, unclear biological plausibility, and was not replicated in a similar RCT. These limitations preclude the drawing of any accurate conclusions on the frequency of adverse events from IAP treatment for neonatal GBS disease prevention. Larger, better quality, and longer studies are needed to provide estimates of adverse events from IAP treatment for neonatal GBS disease prevention.

## Additional file

Additional file 1: The Additional file contains three items. The first shows the search strategy used for Medline that was adapted for other databases; the second lists the studies that were excluded (*n* = 227) at the full text stage with the reasons for their exclusion; and the third is **Table S1.** that summarises all of the included studies, their characteristics, and their findings. (DOCX 225 kb)

## Abbreviations

CFU: Colony forming units
CI: Confidence intervals
*E. coli*: *Escherichia coli*
EOGBS: Early-onset neonatal group B *Streptococcus* disease
GBS: Group B *Streptococcus*
IAP: Intrapartum antibiotic prophylaxis
OR: Odds ratio
RCT: Randomised controlled trial
RD: Risk difference
RoB: Risk of Bias
RoBANS: Risk of bias assessment tool for nonrandomised studies
RR: Relative risk
*S. aureus*: *Staphylococcus aureus*
*S. pneumoniae*: *Streptococcus pneumoniae*
UK: United Kingdom
US: United States of America
