# Supplementary material for: Adverse events in women and children who have received intrapartum antibiotic prophylaxis treatment: a systematic review
Source: BMC Pregnancy Childbirth. 2017 Jul 26;17:247. doi: 10.1186/s12884-017-1432-3 (PMC5530570; doi:10.1186/s12884-017-1432-3)
Supplement: Additional file 1: — The Additional file contains three items. The first shows the search strategy used for Medline that was adapted for other databases; the second lists the studies that were excluded (n = 227) at the full text stage with the reasons for their exclusion; and the third is Table S1. that summarises all of the included studies, their characteristics, and their findings. (DOCX 225 kb) [file 12884_2017_1432_MOESM1_ESM.docx]

**Adverse events in women and children who have received intrapartum antibiotic prophylaxis treatment: A systematic review**

**Supplementary material**

**Search strategy for Medline, adapted for other databases**

| **No.** | **Searches** |
| --- | --- |
| 1 | exp Parturition/ |
| 2 | exp Labor, Obstetric/ |
| 3 | exp Delivery, Obstetric/ |
| 4 | exp Obstetric Labor Complications/ |
| 5 | exp Maternal Health Services/ |
| 6 | (labour or labor).ab,ti,tw. |
| 7 | (intrapartum* or intra-partum*).ab,ti,tw. |
| 8 | "birth* ".ab,ti,tw. |
| 9 | "matern* ".ab,ti,tw. |
| 10 | 1 or 2 or 3 or 4 or 5 or 6 or 7 or 8 or 9 |
| 11 | "prophyla*".ab,ti,tw. |
| 12 | exp Penicillins/ |
| 13 | exp Erythromycin/ |
| 14 | exp Clindamycin/ |
| 15 | exp Cefazolin/ |
| 16 | "penicillin*".ab,ti,tw. |
| 17 | "erythromycin*".ab,ti,tw. |
| 18 | "clindamycin*".ab,ti,tw. |
| 19 | "cefazolin*".ab,ti,tw. |
| 20 | "ampicillin*".ab,ti,tw. |
| 21 | "vancomycin*".ab,ti,tw. |
| 22 | exp Vancomycin/ |
| 23 | 12 or 13 or 14 or 15 or 16 or 17 or 18 or 19 or 20 or 21 or 22 |
| 24 | 11 and 23 |
| 25 | exp Antibiotic Prophylaxis/ |
| 26 | exp Patient Harm/ |
| 27 | exp Product Surveillance, Postmarketing/ |
| 28 | exp Adverse Drug Reaction Reporting Systems/ |
| 29 | exp Clinical Trials, Phase IV as Topic/ |
| 30 | exp Poisoning/ |
| 31 | exp Substance-Related Disorders/ |
| 32 | exp "Drug-Related Side Effects and Adverse Reactions"/ |
| 33 | exp abnormalities, drug induced/ |
| 34 | exp Drug Monitoring/ |
| 35 | exp Drug Hypersensitivity/ |
| 36 | exp Postoperative Complications/ |
| 37 | exp Intraoperative Complications/ |
| 38 | (toxicity or complication* or noxious or tolerability).ab,ti,tw. |
| 39 | (safe or safety).ab,ti,tw. |
| 40 | "side effect*".ab,ti,tw. |
| 41 | ((adverse or undesirable or harms* or serious or toxic) adj3 (effect* or reaction* or event* or outcome*)).ab,ti,tw. |
| 42 | (ae or to or po or co).fs. |
| 43 | exp Drug Resistance/ |
| 44 | exp Microbiota/ |
| 45 | exp Anxiety/co, de [Complications, Drug Effects] |
| 46 | exp Anaphylaxis/ci, co, de [Chemically Induced, Complications, Drug Effects] |
| 47 | exp Overweight/ci, co, de [Chemically Induced, Complications, Drug Effects] |
| 48 | exp Asthma/ci, co, de [Chemically Induced, Complications, Drug Effects] |
| 49 | exp Autistic Disorder/ci, co [Chemically Induced, Complications] |
| 50 | "autis*".ab,ti,tw. |
| 51 | "diabet*".ab,ti,tw. |
| 52 | "obes*".ab,ti,tw. |
| 53 | asthma.ab,ti,tw. |
| 54 | anxiety.ab,ti,tw. |
| 55 | (resistance or resistant).ab,ti,tw. |
| 56 | (microbiome or microbiota).ab,ti,tw. |
| 57 | "anaphyla*".ab,ti,tw. |
| 58 | (overweight or over-weight).ab,ti,tw. |
| 59 | exp Clostridium difficile/de [Drug Effects] |
| 60 | exp Diarrhea/ci, co, po [Chemically Induced, Complications, Poisoning] |
| 61 | ("Clostridium difficile" or "c. diff" or "c. difficile").ab,ti,tw. |
| 62 | (Antibiotic-associated diarrhoea or Antibiotic-associated diarrhea or Antibiotic associated diarrhoea or Antibiotic associated diarrhea).ab,ti,tw. |
| 63 | exp Bacterial Infections/ci, co [Chemically Induced, Complications] |
| 64 | exp Sepsis/ci, co, to [Chemically Induced, Complications, Toxicity] |
| 65 | exp "Length of Stay"/ |
| 66 | exp Skin Diseases/ci, co, to [Chemically Induced, Complications, Toxicity] |
| 67 | exp Respiratory Tract Diseases/ci, co, de [Chemically Induced, Complications, Drug Effects] |
| 68 | exp Cerebral Palsy/ci, co [Chemically Induced, Complications] |
| 69 | length of stay.ab,ti,tw. |
| 70 | (respiratory illness* or respiratory disease*).ab,ti,tw. |
| 71 | cerebral palsy.ab,ti,tw. |
| 72 | (Neonatal Necrotising Enterocolitis or Neonatal Necrotizing Enterocolitis or nec).ab,ti,tw. |
| 73 | exp Candidiasis/ci, co [Chemically Induced, Complications] |
| 74 | exp Enterocolitis, Necrotizing/ci, co [Chemically Induced, Complications] |
| 75 | (yeast infection* or Candidiasis).ab,ti,tw. |
| 76 | (suprainfection* or supra-infection*).ab,ti,tw. |
| 77 | exp Methicillin-Resistant Staphylococcus aureus/de [Drug Effects] |
| 78 | exp Vancomycin-Resistant Enterococci/de [Drug Effects] |
| 79 | exp Inflammatory Bowel Diseases/ci, co [Chemically Induced, Complications] |
| 80 | (Inflammatory bowel disease* or Crohn's disease* or Ulcerative colitis).ab,ti,tw. |
| 81 | exp "Growth and Development"/de [Drug Effects] |
| 82 | (Meticillin-resistant Staphylococcus aureus or Methicillin-resistant Staphylococcus aureus or Meticillin resistant Staphylococcus aureus or Methicillin resistant Staphylococcus aureus or MRSA).ab,ti,tw. |
| 83 | (skin disease* or dematologic* disease* or skin condition* or dematologic* condition*).ab,ti,tw. |
| 84 | (Vancomycin-resistant Enterococci or Vancomycin resistant Enterococci or VRE).ab,ti,tw. |
| 85 | (Extended Spectrum Beta-lactamase or Extended Spectrum Beta lactamase or ESBL).ab,ti,tw. |
| 86 | (Carbapenem-resistant Organism or Carbapenem resistant Organism or CRO).ab,ti,tw. |
| 87 | "antibiotic*".ab,ti,tw. |
| 88 | exp Diabetes Mellitus/ci [Chemically Induced] |
| 89 | (growth adj2 develop*).ab,ti,tw. |
| 90 | 11 and 87 |
| 91 | 24 or 25 or 90 |
| 92 | 26 or 27 or 28 or 29 or 30 or 31 or 32 or 33 or 34 or 35 or 36 or 37 or 38 or 39 or 40 or 41 or 42 or 43 or 44 or 45 or 46 or 47 or 48 or 49 or 50 or 51 or 52 or 53 or 54 or 55 or 56 or 57 or 58 or 59 or 60 or 61 or 62 or 63 or 64 or 65 or 66 or 67 or 68 or 69 or 70 or 71 or 72 or 73 or 74 or 75 or 76 or 77 or 78 or 79 or 80 or 81 or 82 or 83 or 84 or 85 or 86 or 88 or 89 |
| 93 | 10 and 91 and 92 |
| 94 | limit 93 to (english language and humans) |

**Full text studies excluded (n=227), with reason**

| **Reference** | **Reason for exclusion** |
| --- | --- |
| 1. Aard LA, Saed F. Low-incidence cesarean section: 12-year experience. Mayo Clin Proc 1975; 50(7): 365-9. | Prophylaxis for Caesarean section |
| 1. Accordino, F., et al. (2016). Risk factors for cerebral palsy in PPROM and preterm delivery with intact membranes<sup>*</sup>. Journal of Maternal-Fetal and Neonatal Medicine 29(23): 3854-3859. | >10% had symptoms in labour (chorioamnionitis) |
| 1. Adeniran AS, Aboyeji AP, Fawole AA, Adesiyun OO, Saidu R. Role of Risk-Based Approach in the Prevention of Vertical Transmission of Neonatal Sepsis. Niger Postgrad Med J 2015; 22(2): 88-92. | >10% had symptoms in labour (intrapartum fever) |
| 1. Andrews WW, Hauth JC, Cliver SP, Savage K, Goldenberg RL. Randomized clinical trial of extended spectrum antibiotic prophylaxis with coverage for Ureaplasma urealyticum to reduce post-cesarean delivery endometritis. Obstet Gynecol 2003; 101(6): 1183-9. | Prophylaxis for Caesarean section |
| 1. Anonymous. Prophylactic antibiotics in caesarean section. Br Med J 1973; 2(5868): 675-6. | Consensus statement |
| 1. Anonymous. Obesity in pregnancy. Obstet Gynecol 2015; 126(6): e112-e26. | Review |
| 1. Anteby SO, Birkenfeld A, Weinstein D. Post cesarean section urinary tract infections, risk factors and prophylactic antibiotic treatment. Clin Exp Obstet Gynecol 1984; 11(4): 161-4. | Prophylaxis for Caesarean section |
| 1. Apgar BS, Greenberg G, Yen G. Prevention of group B streptococcal disease in the newborn. Am Fam Physician 2005; 71(5): 903-10. | Review |
| 1. Apuzzio JJ, Ganesh VV, Pelosi MA, Frisoli G. The effect of prophylactic antibiotics on risk factors for endomyometritis in adolescent patients undergoing cesarean section. Journal of adolescent health care : official publication of the Society for Adolescent Medicine, 1984. | Prophylaxis for Caesarean section |
| 1. Ayangade O. Antibiotic prophylaxis in high-risk obstetrics. J Natl Med Assoc 1977; 69(11): 793-5. | Unable to identify timing of antibiotics |
| 1. Ayangade O. Long vs short-course antibiotic prophylaxis in cesarean section: a comparative clinical study. J Natl Med Assoc 1979; 71(1): 71-3. | Prophylaxis for Caesarean section |
| 1. Azad, M. B., et al. (2016). Impact of maternal intrapartum antibiotics, method of birth and breastfeeding on gut microbiota during the first year of life: A prospective cohort study. BJOG: An International Journal of Obstetrics and Gynaecology 123(6): 983-993 | >10% had symptoms in labour (prolonged rupture of membranes) |
| 1. Battarino O, Battarino A. [Short-term antibiotic prophylaxis in cesarean section]. Minerva ginecologica, 1988. | Full text not in English |
| 1. Beattie PG, Rings TR, Hunter MF, Lake Y. Risk factors for wound infection following caesarean section. Aust N Z J Obstet Gynaecol 1994; 34(4): 398-402. | Prophylaxis for Caesarean section |
| 1. Benigno BB, Ford LC, Lawrence WD, Ledger WJ, Ling FW, McNeeley SG. A double-blind, controlled comparison of piperacillin and cefoxitin in the prevention of postoperative infection in patients undergoing cesarean section. Surg Gynecol Obstet, 1986. | Prophylaxis for Caesarean section |
| 1. Benjamin DK, Stoll BJ, Gantz MG, et al. Neonatal Candidiasis: Epidemiology, Risk Factors, and Clinical Judgment. Pediatrics 2010; 126(4): E865-E73. | Unable to distinguish intrapartum antibiotics with other timings |
| 1. Berardi A, Rossi C, Creti R, et al. Group B Streptococcal colonization in 160 mother-baby pairs: A prospective cohort study. J Pediatr 2013; 163(4): 1099-104.e1. | No data on adverse events |
| 1. Berardi, A., et al. (2016). "The burden of early-onset sepsis in Emilia-Romagna (Italy): a 4-year, population-based study." Journal of Maternal-Fetal and Neonatal Medicine 29(19): 3126-3131. | >10% had symptoms in labour (prolonged rupture of membranes) |
| 1. Berkeley AS, Hirsch JC, Freedman KS, Ledger WJ. Cefotaxime for cesarean section prophylaxis in labor. Intravenous administration vs. lavage. Journal of Reproductive Medicine for the Obstetrician and Gynecologist 1990; 35(3): 214-8. | Prophylaxis for Caesarean section |
| 1. Bibi M, Megdiche H, Ghanem H, et al. [Antibiotic prophylaxis in a priori cesarean sections without a high risk of infection. Experiences of a Tunisian maternity department]. Journal de gynécologie, obstétrique et biologie de la reproduction, 1994. | Full text not in English |
| 1. Birkenfeld A, Anteby SO. The effect of ampicillin and colistin on post-Caesarean section endometritis with identification of possible risk factors. Aust N Z J Obstet Gynaecol 1983; 23(4): 204-7. | Prophylaxis for Caesarean section |
| 1. Block BS, Mercer LJ, Ismail MA, Moawad AH. Clostridium difficile-associated diarrhea follows perioperative prophylaxis with cefoxitin. Am J Obstet Gynecol 1985; 153(8): 835-8. | >10% prophylaxis for Caesarean section |
| 1. Boothby R, Benrubi G, Ferrell E. Comparison of intravenous cefoxitin prophylaxis with intraoperative cefoxitin irrigation for the prevention of post-cesarean-section endometritis. Journal of Reproductive Medicine for the Obstetrician and Gynecologist 1984; 29(11): 830-2. | Prophylaxis for Caesarean section |
| 1. Bourgeois FJ, Pinkerton JA, Andersen W, Thiagarajah S. Antibiotic irrigation prophylaxis in the high-risk cesarean section patient. Am J Obstet Gynecol 1985; 153(2): 197-201. | Prophylaxis for Caesarean section |
| 1. Boyer KM, Gotoff SP. Prevention of early-onset neonatal group B streptococcal disease with selective intrapartum chemoprophylaxis. N Engl J Med 1986; 314(26): 1665-9. | >10% participants had symptoms in labour (maternal fever) |
| 1. Bromiker R, Ernest N, Meir MB, et al. Correlation of bacterial type and antibiotic sensitivity with maternal antibiotic exposure in early-onset neonatal sepsis. Neonatology 2013; 103(1): 48-53. | >10% participants had symptoms in labour (maternal fever) |
| 1. Brown J, Thompson M, Sinnya S, et al. Pre-incision antibiotic prophylaxis reduces the incidence of post-caesarean surgical site infection. J Hosp Infect 2013; 83(1): 68-70. | Prophylaxis for Caesarean section |
| 1. Brozanski BS, Jones JG, Krohn MA, Sweet RL. Effect of a screening-based prevention policy on prevalence of early-onset group B streptococcal sepsis. Obstet Gynecol 2000; 95(4): 496-501. | No data on adverse events |
| 1. Buchholz NP, Daly-Grandeau E, Huber-Buchholz MM. Urological complications associated with caesarean section. Eur J Obstet Gynecol Reprod Biol 1994; 56(3): 161-3. | Prophylaxis for Caesarean section |
| 1. Busowski JD, Porter KB, Pendergraft S, O'Brien WF, Vodra J. Antibiotic prophylaxis for Cesarean delivery: a randomized trial of cefotetan, ampicillin-sulbactam and ciprofloxacin. Prenat Neonatal Med 2000; 5(6): 357-62. | Prophylaxis for Caesarean section |
| 1. Carlson C, Duff P. Antibiotic prophylaxis for cesarean delivery: is an extended-spectrum agent necessary? Obstet Gynecol 1990; 76(3 Pt 1): 343-6. | Prophylaxis for Caesarean section |
| 1. Carney E. Antibiotic prophylaxis in obstetrics and gynecology. J Med Assoc State Ala 1975; 44(9): 493-4, 9. | Review |
| 1. Cassidy-Bushrow AE, Sitarik A, Levin AM, et al. Maternal group B Streptococcus and the infant gut microbiota. J Dev Orig Health Dis 2016; 7(1): 45-53. | >10% neonates received antibiotics after birth |
| 1. Chan AC, Leung AK, Chin RK, Chang AM. Single dose prophylactic antibiotics in caesarean sections. Aust N Z J Obstet Gynaecol 1989; 29(2): 107-9. | Prophylaxis for Caesarean section |
| 1. Chang PL, Newton ER. Predictors of antibiotic prophylactic failure in post-cesarean endometritis. Obstet Gynecol 1992; 80(1): 117-22. | Prophylaxis for Caesarean section |
| 1. Chantharojwong P. An efficacy study of ampicillin versus cefazolin prophylaxis in patients undergoing cesarean section. J Med Assoc Thai 1993; 76(3): 165-70. | Prophylaxis for Caesarean section |
| 1. Chimura T. The efficacy of ceftriaxone administered for prophylaxis of postoperative infection and infectious diseases in obstetrics and gynecology. J Chemother 1989; 1(4 Suppl): 1039-41. | Antibiotics administered after birth |
| 1. Chittacharoen A, Manonai J, Suthutvoravut S, Phaupradit W. Single-dose amoxycillin-clavulanic acid vs. ampicillin prophylaxis in emergency cesarean section. International Journal of Gynecology and Obstetrics 1998; 62(3): 249-54. | Prophylaxis for Caesarean section |
| 1. Conover WB, Moore TR. Comparison of irrigation and intravenous antibiotic prophylaxis at cesarean section. Obstet Gynecol 1984; 63(6): 787-91. | Prophylaxis for Caesarean section |
| 1. Conturso R, Valsecchi A, De Lalla F. Evaluation of mezlocillin versus placebo as a prophylactic agent in cesarean section. Chemioterapia 1987; 6(2 Suppl): 611-3. | Prophylaxis for Caesarean section |
| 1. Currier JS, Tosteson TD, Platt R. Cefazolin compared with cefoxitin for cesarean section prophylaxis: the use of a two-stage study design. J Clin Epidemiol 1993; 46(7): 625-30. | Prophylaxis for Caesarean section |
| 1. Cyrkowicz A, Rytwińska E, Nytko J, Słowińska-Zabówka M. [Preparation for delivery in patients with missed labor considering low-dose heparin and prostaglandins]. Przegla̧d lekarski, 1996. | Full text not in English |
| 1. D'Angelo LJ, Sokol RJ. Short- versus long-course prophylactic antibiotic treatment in Cesarean section patients. Obstet Gynecol 1980; 55(5): 583-6. | Prophylaxis for Caesarean section |
| 1. Daley AJ, Isaacs D. Ten-year study on the effect of intrapartum antibiotic prophylaxis on early onset group B streptococcal and Escherichia coli neonatal sepsis in Australasia. Pediatr Infect Dis J 2004; 23(7): 630-4. | Population level ecological study |
| 1. Dashefsky B. Prophylaxis against neonatal group B streptococcal disease. Pediatr Infect Dis J 1990; 9(2): 147-9. | Letter |
| 1. Davey P. Antimicrobial prophylaxis for caesarean section - the unanswered questions. Journal of Obstetrics and Gynaecology 1992; 12(SUPPL. 1): S21-S3. | Review |
| 1. De Luca C, Buono N, Santillo V, et al. Screening and management of maternal colonization with Streptococcus agalactiae: an Italian cohort study. J Matern-Fetal Neonatal Med 2016; 29(6): 911-5. | No data on adverse events |
| 1. Decavalas G, Maroulis G, Papaioannou C, Papapetropoulou M. Comparative study of ceftriaxone versus cefamandole for pre-operative prophylaxis of infections in patients undergoing cesarean section or vaginal hysterectomy. J Chemother 1989; 1(4 Suppl): 1048-50. | Prophylaxis for Caesarean section |
| 1. Dlamini LD, Sekikubo M, Tumukunde J, et al. Antibiotic prophylaxis for caesarean section at a Ugandan hospital: a randomised clinical trial evaluating the effect of administration time on the incidence of postoperative infections. BMC Pregnancy & Childbirth 2015; 15: 91. | Prophylaxis for Caesarean section |
| 1. Donnenfeld AE, Otis C, Weiner S. Antibiotic prophylaxis in cesarean section. Comparison of intrauterine lavage and intravenous administration. The Journal of reproductive medicine, 1986. | Prophylaxis for Caesarean section |
| 1. Duff P, Park RC. Antibiotic prophylaxis for cesarean section in a military population. Mil Med 1980; 145(6): 377-81. | Prophylaxis for Caesarean section |
| 1. Dumas AM, Girard R, Ayzac L, et al. Effect of intrapartum antibiotic prophylaxis against group B streptococcal infection on comparisons of rates of endometritis and urinary tract infection in multicenter surveillance. Infect Control Hosp Epidemiol 2008; 29(4): 327-32. | No data on adverse events |
| 1. Easmon CSF, Hastings MJG, Deeley J. The effect of intrapartum chemoprophylaxis on the vertical transmission of group B streptococci. Br J Obstet Gynaecol 1983; 90(7): 633-5. | No data on adverse events |
| 1. Ecker KL, Donohue PK, Kim KS, Shepard JA, Aucott SW. The impact of group B streptococcus prophylaxis on late-onset neonatal infections. J Perinatol 2013; 33(3): 206-11. | >10% participants had symptoms in labour (prolonged rupture of membranes) |
| 1. Edwards RK, Clark P, Sistrom CL, Duff P. Intrapartum antibiotic prophylaxis 1: Relative effects of recommended antibiotics on gram-negative pathogens. Obstet Gynecol 2002; 100(3): 534-9. | >10% antibiotics received antibiotics before labour |
| 1. Elliott JP, Flaherty JF. Comparison of lavage or intravenous antibiotics at cesarean section. Obstet Gynecol, 1986. | Prophylaxis for Caesarean section |
| 1. Elliott JP, Freeman RK, Dorchester W. Short versus long course of prophylactic antibiotics. Am J Obstet Gynecol 1982; 143(7): 740-4. | >10% prophylaxis for Caesarean section |
| 1. Elyan A, Mahran M, el-Maraghy M, Abou-Seeda M. Prophylactic intravenous metronidazole in cesarean section. Chemioterapia 1984; 3(1): 67-70. | Prophylaxis for Caesarean section |
| 1. Engel K, Karschnia R. Bacterial flora changes resulting from antimicrobial treatment. Journal of Obstetrics and Gynaecology 1986; 6(SUPPL. 1): S6-S8. | >10% prophylaxis for Caesarean section |
| 1. Engel K, Karschnia R, Rauch U, Amir B. Efficacy of a high dosage short course prophylactic treatment for postoperative infection complications in cesarean section - using a combination of mezlocillin and oxacillin (Optocillin). Chemioterapia 1982; 1(4 Suppl.): No. 326. | Prophylaxis for Caesarean section |
| 1. Escobedo Lobatón JM, Rodríguez Hinojosa DE, Kistner Garza AM, Benavides de Anda L. [Prophylactic use of antibiotics in cesarean section]. Ginecología y obstetricia de México, 1991. | Full text not in English |
| 1. Faro S, Cox SM, Phillips L, Baker J. Influence of antibiotic prophylaxis on vaginal microflora. Journal of Obstetrics and Gynaecology 1986; 6(SUPPL. 1): S4-S6. | >10% received antibiotics for Caesarean section |
| 1. Faro S, Martens MG, Hammill HA, Riddle G, Tortolero G. Antibiotic prophylaxis: is there a difference? Am J Obstet Gynecol 1990; 162(4): 900-7; discussion 7-9. | >10% prophylaxis for Caesarean section |
| 1. Farret TCF, Dalle J, da Silva Monteiro V, Riche CVW, Antonello VS. Risk factors for surgical site infection following cesarean section in a Brazilian Women's Hospital: A case-control study. Braz J Infect Dis 2015; 19(2): 113-7. | Prophylaxis for Caesarean section |
| 1. Fedele L, Acaia B, Marchini M, Baglioni A, Frigoli A, De Pascale A. Cefotetan and ceftriaxone for single-dose prophylaxis in cesarean section. J Chemother 1989; 1(4 Suppl): 1042-3. | Prophylaxis for Caesarean section |
| 1. Fejgin MD, Markov S, Goshen S, Segal J, Arbel Y, Lang R. Antibiotic for cesarean section: the case for 'true' prophylaxis. Int J Gynaecol Obstet 1993; 43(3): 257-61. | Prophylaxis for Caesarean section |
| 1. Felton DJ, Williams JD. Prophylactic ampicillin the the surgical induction of labour. J Obstet Gynaecol Br Commonw 1967; 74(6): 862-7. | Antibiotics administered before labour |
| 1. Fonseca SNS, Sofia MH, Quintana S, Nogueira FDS, Levin AS. Successful control program to implement the appropriate antibiotic prophylaxis for cesarean section. Rev Inst Med Trop Sao Paulo 2008; 50(2): 79-82. | Prophylaxis for Caesarean section |
| 1. Ford LC. Cost of antibiotic prophylaxis in cesarean section. Drug Intell Clin Pharm 1986; 20(7-8): 592-3. | Prophylaxis for Caesarean section |
| 1. Ford LC, Tabsh K, Lebherz TB. Use of antibiotics for prophylaxis with caesarean section. Journal of Obstetrics and Gynaecology 1986; 6(SUPPL. 1): S68-S70. | Prophylaxis for Caesarean section |
| 1. Francis C, Mumford M, Strand ML, Moore ES, Strand EA. Timing of prophylactic antibiotic at cesarean section: a double-blinded, randomized trial. J Perinatol 2013; 33(10): 759-62. | Prophylaxis for Caesarean section |
| 1. Freeman GM. The efficacy of prophylactic antibiotics in high-risk patients undergoing cesarean section. J-Am-Osteopath-Assoc, 1982. | Prophylaxis for Caesarean section |
| 1. Galask RP, Weiner C, Petzold CR. Comparison of single-dose cefmetazole and cefotetan prophylaxis in women undergoing primary caesarean section. J Antimicrob Chemother 1989; 23 Suppl D: 105-8. | Prophylaxis for Caesarean section |
| 1. Gall SA. The efficacy of prophylactic antibiotics in cesarean section. Am J Obstet Gynecol, 1979. | Prophylaxis for Caesarean section |
| 1. Gall SA, Hill GB. Single-dose versus multiple-dose piperacillin prophylaxis in primary cesarean operation. Am J Obstet Gynecol 1987; 157(2): 502-6. | S Prophylaxis for Caesarean section |
| 1. Gerard P, Verghote-D'Hulst M, Bachy A, Duhaut G. Group B streptococcal colonization of pregnant women and their neonates. Epidemiological study and controlled trial of prophylactic treatment of the newborn. Acta Paediatr Scand, 1979. | Antibiotics administered after birth |
| 1. Gerber B, Retzke F, Wilken H. [Effectiveness of perioperative preventive use of antibiotics with ampicillin/gentamycin or cefotiam in abdominal cesarean section]. Zentralblatt für Gynäkologie, 1989. | Full text not in English |
| 1. Gerstner G, Kofler E, Huber J. [Perioperative metronidazole-prophylaxis for cesarian section (author's transl]. Zeitschrift für Geburtshilfe und Perinatologie, 1980. | Full text not in English |
| 1. Ghuman M, Rohlandt D, Joshy G, Lawrenson R. Post-caesarean section surgical site infection: Rate and risk factors. N Z Med J 2011; 124(1339): 32-6. | Prophylaxis for Caesarean section |
| 1. Gibbs RS, DeCherney AH, Schwarz RH. Prophylactic antibiotics in cesarean section: a double-blind study. Am J Obstet Gynecol 1972; 114(8): 1048-53. | Prophylaxis for Caesarean section |
| 1. Gibbs RS, Hunt JE, Schwarz RH. A follow-up study on prophylactic antibiotics in cesarean section. Am J Obstet Gynecol 1973; 117(3): 419-22. | Prophylaxis for Caesarean section |
| 1. Gibbs RS, Weinstein AJ. Bacteriologic effects of prophylactic antibiotics in cesarean section. Am J Obstet Gynecol, 1976. | Prophylaxis for Caesarean section |
| 1. Gidiri MF, Ziruma A. A randomized clinical trial evaluating prophylactic single-dose vs prolonged course of antibiotics for caesarean section in a high HIV-prevalence setting. J Obstet Gynaecol 2014; 34(2): 160-4. | Prophylaxis for Caesarean section |
| 1. Giuliani B, Periti E, Mecacci F. Antimicrobial prophylaxis in obstetric and gynecological surgery. J Chemother 1999; 11(6): 577-80. | Surgical prophylaxis |
| 1. Glasgow TS, Speakman M, Firth S, James B, Byington CL, Young PC. Clinical and economic outcomes for term infants associated with increasing administration of antibiotics to their mothers. Paediatr Perinat Epidemiol 2007; 21(4): 338-46. | Unclear when antibiotic was given |
| 1. Gonen R, Samberg I, Levinski R. Effect of irrigation or intravenous antibiotic prophylaxis on infectious morbidity at cesarean section. Obstet Gynecol 1986; 67(4): 545-8. | Prophylaxis for Caesarean section |
| 1. Gong SP, Guo HX, Zhou HZ, Chen L, Yu YH. Morbidity and risk factors for surgical site infection following cesarean section in Guangdong Province, China. J Obstet Gynaecol Res 2012; 38(3): 509-15. | Prophylaxis for Caesarean section |
| 1. Gonik B, Shannon RL, Shawar R, Costner M, Seibel M. Why patients fail antibiotic prophylaxis at cesarean delivery: histologic evidence for incipient infection. Obstet Gynecol 1992; 79(2): 179-84. | Prophylaxis for Caesarean section |
| 1. Gordon HR, Phelps D, Blanchard K. Prophylactic cesarean section antibiotics: maternal and neonatal morbidity before or after cord clamping. Obstet Gynecol 1979; 53(2): 151-6. | Prophylaxis for Caesarean section |
| 1. Gordon SF, Russell J. A randomized controlled study comparing ceftizoxime, cefamandole, and cefoxitin in obstetric and gynecological surgery: A preliminary report. J Antimicrob Chemother 1982; 10(Suppl. C): 289-92. | Surgical prophylaxis |
| 1. Green SL, Sarubbi FA, Jr., Bishop EH. Prophylactic antibiotics in high-risk cesarean section. Obstet Gynecol 1978; 51(5): 569-72. | Prophylaxis for Caesarean section |
| 1. Gronlund MM, Lehtonen OP, Eerola E, Kero P. Fecal microflora in healthy infants born by different methods of delivery: Permanent changes in intestinal flora after cesarean delivery. J Pediatr Gastroenterol Nutr 1999; 28(1): 19-25. | >10% participants had elective caesarean section |
| 1. Grossman Donowitz L, Norris SM. The efficacy of antibiotic prophylaxis in the prevention of post-cesarean section endometritis. *Infect Control* 1985; 6(5): 189-93. | Prophylaxis for Caesarean section |
| 1. Habib FA. Incidence of post cesarean section wound infection in a tertiary hospital, Riyadh, Saudi Arabia. Saudi Med J 2002; 23(9): 1059-63. | Prophylaxis for Caesarean section |
| 1. Haesslein HC, Goodlin RC. Extraperitoneal cesarean section revisited. Obstet Gynecol 1980; 55(2): 181-3. | Prophylaxis for Caesarean section |
| 1. Hager WD, Rapp RP, Billeter M, Bradley BB. Choice of antibiotic in nonelective cesarean section. Antimicrob Agents Chemother 1991; 35(9): 1782-4. | Prophylaxis for Caesarean section |
| 1. Harger JH, English DH. Selection of patients for antibiotic prophylaxis in cesarean sections. Am J Obstet Gynecol 1981; 141(7): 752-8. | Prophylaxis for Caesarean section |
| 1. Harries MJ, McIntyre SJ, Kingston TP. Co-amoxiclav-induced acute generalized exanthematous pustulosis confirmed by patch testing. Contact Dermatitis 2006; 55(6): 372. | Case report |
| 1. Heilmann L, Tauber PF. [Short-term prevention with cefoxitin in cesarean section]. Geburtshilfe Frauenheilkd, 1984. | Full text not in English |
| 1. Iqbal R, Intsar A, Khurshid S, Manzoor T, Shehbaz S. Single dose antibiotic prophylaxis in emergency caesarean section. Pakistan Journal of Medical and Health Sciences 2012; 6(1): 77-80. | Prophylaxis for Caesarean section |
| 1. Itskovitz J, Paldi E, Katz M. The effect of prophylactic antibiotics on febrile morbidity following cesarean section. Obstet Gynecol 1979; 53(2): 162-5. | Prophylaxis for Caesarean section |
| 1. Jaffe R, Altaras M, Cohen I, Ben-Aderet N. Single-dose mezlocillin prophylaxis in emergency cesarean section. Clin Ther 1985; 7(4): 507-11. | Prophylaxis for Caesarean section |
| 1. Jaffe R, Altaras M, Loebel R, Ben-Aderet N. Single- versus multiple-dose mezlocillin prophylaxis in emergency cesarean section. Chemotherapy 1986; 32(2): 173-7. | Prophylaxis for Caesarean section |
| 1. Jaffe R, Loebel R, Altaras M, Ben Aderet N. Perioperative mezlocillin prophylaxis in cesarean section. Clin Ther 1984; 6(4): 467-74. | Prophylaxis for Caesarean section |
| 1. Jakobi P, Weissman A, Sigler E, Margolis K, Zimmer EZ. Post-cesarean section febrile morbidity. Antibiotic prophylaxis in low-risk patients. J Reprod Med 1994; 39(9): 707-10. | Prophylaxis for Caesarean section |
| 1. Jakobi P, Weissman A, Zimmer EZ, Paldi E. Single-dose cefazolin prophylaxis for cesarean section. Am J Obstet Gynecol 1988; 158(5): 1049-52. | Prophylaxis for Caesarean section |
| 1. Jenícek J, Fait T, Jedlicková A, Zivný J. [Antibiotic prophylaxis of infectious complications in cesarean section--prospective study]. Ceská gynekologie / Ceská lékarská spolecnost J Ev Purkyne, 1999. | Full text not in English |
| 1. Kaimal AJ, Zlatnik MG, Cheng YW, et al. Effect of a change in policy regarding the timing of prophylactic antibiotics on the rate of postcesarean delivery surgical-site infections. Am J Obstet Gynecol 2008; 199(3): 310.e1-5. | Prophylaxis for Caesarean section |
| 1. Kamilya G, Seal SL, Mukherji J, Roy H, Bhattacharyya SK, Hazra A. A Randomized controlled trial comparing two different antibiotic regimens for prophylaxis at cesarean section. Journal of Obstetrics and Gynecology of India 2012; 62(1): 35-8. | Prophylaxis for Caesarean section |
| 1. Katz VL, Moos MK, Cefalo RC, Thorp Jr JM, Bowes Jr WA, Wells SD. Group B streptococci: Results of a protocol of antepartum screening and intrapartum treatment. Am J Obstet Gynecol 1994; 170(2): 521-6 | >10% had premature rupture of membranes |
| 1. Kayihura V, Osman NB, Bugalho A, Bergström S. Choice of antibiotics for infection prophylaxis in emergency cesarean sections in low-income countries: a cost-benefit study in Mozambique. Acta Obstet Gynecol Scand, 2003. | Prophylaxis for Caesarean section |
| 1. Kittur ND, McMullen KM, Russo AJ, Ruhl L, Kay HH, Warren DK. Long-term effect of infection prevention practices and case mix on cesarean surgical site infections. Obstet Gynecol 2012; 120(2 Pt 1): 246-51. | Prophylaxis for Caesarean section |
| 1. Knottenbelt JD. Antibiotic prophylaxis against sepsis after caesarean section. Cent Afr J Med 1979; 25(7): 148-50. | Prophylaxis for Caesarean section |
| 1. Krasnodebski J, Stolecki M. [A single dose of antibiotic--as a prophylaxis during cesarean section]. Ginekol Pol, 1997. | Full text not in English |
| 1. Kreutner AK, Bene VE, Delamar D, Bodden JL, Loadholt CB. Perioperative cephalosporin prophylaxis in cesarean section: effect on endometritis in the high-risk patient. Am J Obstet Gynecol, 1979. | Prophylaxis for Caesarean section |
| 1. Kreutner AK, Bene VE, Delamar D, Huguley V, Harmon PM, Mitchell KS. Perioperative antibiotic prophylaxis is cesarean section. Obstet Gynecol, 1978. | Prophylaxis for Caesarean section |
| 1. Kunze M, Ziegler A, Fluegge K, Hentschel R, Proempeler H, Berner R. Colonization, serotypes and transmission rates of group B streptococci in pregnant women and their infants born at a single University Center in Germany. J Perinat Med 2011; 39(4): 417-22. | No data on adverse events |
| 1. Lemus Rocha R, García Gutiérrez LB, Basavilvazo Rodríguez MA, Cruz Avelar A, Peralta Pedrero ML, Hernández Valencia M. [Incidence of infected surgical wound and prophylaxis with cefotaxime in cesarean section]. Ginecología y obstetricia de México, 2005. | Full text not in English |
| 1. Levine EM, Ghai V, Barton JJ, Strom CM. Intrapartum antibiotic prophylaxis increases the incidence of gram-negative neonatal sepsis. Infect Dis Obstet Gynecol 1999; 7(4): 210-3. | >10% had risk factors (preterm delivery, PROM, fever, prior GBS bacteriuria |
| 1. Lewis DF, Otterson WN, Dunnihoo DR. Antibiotic prophylactic uterine lavage in cesarean section: a double-blind comparison of saline, ticarcillin, and cefoxitin irrigation in indigent patients. South Med J 1990; 83(3): 274-6. | Prophylaxis for Caesarean section |
| 1. Long SS. Blame inappropriate implementation for failure of intrapartum antibiotic prophylaxis for group B Streptococcus. J Pediatr 2010; 156(3): A1. | Editorial |
| 1. Louie TJ, Binns BA, Baskett TF, Ross J, Koss J. Cefotaxime, cefazolin, or ampicillin prophylaxis of febrile morbidity in emergency cesarean sections. Clin Ther, 1982. | Prophylaxis for Caesarean section |
| 1. Lyimo FM, Massinde AN, Kidenya BR, Konje ET, Mshana SE. Single dose of gentamicin in combination with metronidazole versus multiple doses for prevention of post-caesarean infection at Bugando Medical Centre in Mwanza, Tanzania: a randomized, equivalence, controlled trial. BMC Pregnancy & Childbirth 2013; 13: 123. | Prophylaxis for Caesarean section |
| 1. Mah MW, Pyper AM, Oni GA, Memish ZA. Impact of antibiotic prophylaxis on wound infection after cesarean section in a situation of expected higher risk. Am J Infect Control 2001; 29(2): 85-8. | Prophylaxis for Caesarean section |
| 1. Matani, C., et al. (2016). "Streptococcus agalactiae: prevalence of antimicrobial resistance in vaginal and rectal swabs in Italian pregnant women." Infezioni in Medicina 24(3): 217-221. | No IAP information/ population level study |
| 1. Mansueto GB, Tomaselli F. [Antibiotic prophylaxis in non-elective cesarean section with single-dose imipenem versus multiple-dose cefotaxime]. Rivista europea per le scienze mediche e farmacologiche = European review for medical and pharmacological sciences = Revue européenne pour les sciences médicales et pharmacologiques, 1989. = | Full text not in English |
| 1. Mathelier AC. A comparison of postoperative morbidity following prophylactic antibiotic administration by combined irrigation and intravenous route or by intravenous route alone during cesarean section. J Perinat Med 1992; 20(3): 177-82. | Prophylaxis for Caesarean section |
| 1. Matorras R, Garcia-Perea A, Madero R, Usandizaga JA. Maternal colonization by group B streptococci and puerperal infection; analysis of intrapartum chemoprophylaxis. Eur J Obstet Gynecol Reprod Biol 1991; 38(3): 203-7. | >10% participants had elective caesarean section |
| 1. Matorras R, Garcia-Perea A, Omenaca F, Diez-Enciso M, Madero R, Usandizaga JA. Intrapartum chemoprophylaxis of early-onset group B streptococcal disease. Eur J Obstet Gynecol Reprod Biol 1991; 40(1): 57-62. | >10% participants had elective caesarean section |
| 1. May SM, Hartz MF, Joshi AY, Park MA. Intrapartum antibiotic exposure for group B Streptococcus treatment did not increase penicillin allergy in children. Annals of Allergy, Asthma and Immunology 2016; 116(2): 134-8. | >10% IAP given for Caesarean section surgery |
| 1. McCowan L, Jackson P. The prophylactic use of metronidazole in caesarean section. N Z Med J 1980; 92(666): 153-5. | Prophylaxis for Caesarean section |
| 1. McGregor JA, French JI, Makowski E. Single-dose cefotetan versus multidose cefoxitin for prophylaxis in cesarean section in high-risk patients. Am J Obstet Gynecol 1986; 154(4): 955-60. | Prophylaxis for Caesarean section |
| 1. Mengist, A., et al. (2016). "Prevalence and antimicrobial susceptibility pattern of anorectal and vaginal group B Streptococci isolates among pregnant women in Jimma, Ethiopia." BMC Research Notes 9: 351. | No IAP information/ population level study |
| 1. Melendez J, Claxton A, Erskine K. MRSA bacteraemia after caesarean section wound infection: when screening is missed and things go wrong. Arch Gynecol Obstet 2012; 285(3): 663-5. | Case report |
| 1. Menson EN, Gilbert RE, Sharland MR. What is the effect of prepartum antimicrobials on neonatal infection? Curr Opin Infect Dis 2004; 17(3): 213-6. | Review |
| 1. Meyer NL, Hosier KV, Scott K, Lipscomb GH. Cefazolin versus cefazolin plus metronidazole for antibiotic prophylaxis at cesarean section. South Med J, 2003. | Prophylaxis for Caesarean section |
| 1. Mihailovic M, Hani A, Klainguti A, Soldini G. Antimicrobial prophylaxis in non-infected patients undergoing abdominal or vaginal hysterectomy or cesarean section. Comparative efficacy of a single preoperative dose of ceftriaxone and of multiple doses of combined amoxicillin plus metronidazole and of amoxicillin alone. J Chemother 1989; 1(4 Suppl): 1029-30. | Prophylaxis for Caesarean section or hysterectomy |
| 1. Mivumbi VN, Little SE, Rulisa S, Greenberg JA. Prophylactic ampicillin versus cefazolin for the prevention of post-cesarean infectious morbidity in Rwanda. Int J Gynaecol Obstet 2014; 124(3): 244-7. | Prophylaxis for Caesarean section |
| 1. Moberg PJ, Schedvins K. Use of cefuroxime in preventing postcesarean infection in high-risk patients. Gynecol Obstet Invest 1989; 28(1): 19-22. | Prophylaxis for Caesarean section |
| 1. Moodley J, Zeeman DJ. Prophylactic and antimicrobial therapy using lincomycin in patients undergoing emergency caesarean section. S Afr Med J 1981; 59(25): 911-3. | Prophylaxis for Caesarean section |
| 1. Moro M, Andrews M. Prophylactic antibiotics in cesarean section. Obstet Gynecol 1974; 44(5): 688-92. | Prophylaxis for Caesarean section |
| 1. Morrison JC, Coxwell WL, Kennedy BS, Schreier PC, Wiser WL, Fish SA. The use of prophylactic antibiotics in patients undergoing cesarean section. Surg Gynecol Obstet, 1973. | Prophylaxis for Caesarean section |
| 1. Mothilal M, Thivya R, Anjalakshi C, Ramesh A, Damodharan N. Comparison of effectiveness of Azithromycin and Cefazolin in post caesarean section infection. International Journal of Pharmacy and Pharmaceutical Sciences 2013; 5(SUPPL 3): 92-4. | Prophylaxis for Caesarean section |
| 1. Newton ER, Prihoda TJ, Gibbs RS. A clinical and microbiologic analysis of risk factors for puerperal endometritis. Obstet Gynecol 1990; 75(3 Pt 1): 402-6. | Prophylaxis for Caesarean section |
| 1. Newton ER, Wallace PA. Effects of prophylactic antibiotics on endometrial flora in women with postcesarean endometritis. Obstet Gynecol 1998; 92(2): 262-8. | Participants had endometritis at the beginning of study |
| 1. Ng NK, Sivalingam N. The role of prophylactic antibiotics in caesarean section--a randomised trial. Med J Malaysia 1992; 47(4): 273-9. | Prophylaxis for Caesarean section |
| 1. Ngoc NTN, Sloan NL, Thach TS, Liem LKB, Winikoff B. Incidence of postpartum infection after vaginal delivery in Viet Nam. J Heatlh Popul Nutr 2005; 23(2): 121-30. | No data on adverse events and antibiotics given after birth |
| 1. Nice C, Feeney A, Godwin P, et al. A prospective audit of wound infection rates after caesarean section in five West Yorkshire hospitals. J Hosp Infect 1996; 33(1): 55-61. | Prophylaxis for Caesarean section |
| 1. Nokiani FA, Akbari H, Rezaei M. Timing of prophylactic antibiotic administration in term cesarean section: A randomized clinical trial. Iranian Journal of Clinical Infectious Diseases 2009; 4(2): 71-6. | Prophylaxis for Caesarean section |
| 1. O'Leary JA, Mullins JH, Andrinopoulos GC. Ampicillin vs. ampicillin-gentamicin prophylaxis in high-risk primary cesarean section. The Journal of reproductive medicine, 1986. | Prophylaxis for Caesarean section |
| 1. Ogasawara KK, Goodwin TM. Efficacy of azithromycin in reducing lower genital Ureaplasma urealyticum colonization in women at risk for preterm delivery. The Journal of maternal-fetal medicine, 1999. | >10% had preterm premature rupture of membranes |
| 1. Ogasawara KK, Murphy Goodwin T. The efficacy of prophylactic erythromycin in preventing vertical transmission of Ureaplasma urealyticum. Am J Perinatol 1997; 14(4): 233-7. | >10% preterm premature rupture of membranes |
| 1. Ognissanti F, Bucciero A, Conturso R, et al. A comparison of mezlocillin and cefotetan in cesarean section prophylaxis: a prospective, randomized study. Preliminary results. J Chemother 1989; 1(4 Suppl): 1030-2. | Prophylaxis for Caesarean section |
| 1. Oliva GC, Fratoni A, Papadia LS, Tartaglia E, Mancuso S. Antibiotic prophylaxis in emergency and elective cesarean section. J Chemother 1989; 1(4 Suppl): 1020-2. | Prophylaxis for Caesarean section |
| 1. Owens SM, Brozanski BS, Meyn LA, Wiesenfeld HC. Antimicrobial Prophylaxis for Cesarean Delivery Before Skin Incision. Obstet Gynecol 2009; 114(3): 573-9. | Prophylaxis for Caesarean section |
| 1. Padilla SL, Spence MR, Beauchamp PJ. Single-dose ampicillin for cesarean section prophylaxis. Obstet Gynecol 1983; 61(4): 463-6. | Prophylaxis for Caesarean section |
| 1. Periti P, Mazzei T, Periti E. Prophylaxis in gynaecological and obstetric surgery: a comparative randomised multicentre study of single-dose cefotetan versus two doses of cefazolin. Chemioterapia : international journal of the Mediterranean Society of Chemotherapy, 1988. | Surgical prophylaxis |
| 1. Persaud RR, Azad MB, Chari RS, et al. Perinatal antibiotic exposure of neonates in Canada and associated risk factors: a population-based study. J Matern-Fetal Neonatal Med 2015; 28(10): 1190-5. | No data on adverse events |
| 1. Peterson CM, Medchill M, Gordon DS, Chard HL. Cesarean prophylaxis: a comparison of cefamandole and cefazolin by both intravenous and lavage routes, and risk factors associated with endometritis. Obstet Gynecol 1990; 75(2): 179-82. | Prophylaxis for Caesarean section |
| 1. Phelan JP, Pruyn SC. Prophylactic antibiotics in cesarean section: a double-blind study of cefazolin. Am J Obstet Gynecol 1979; 133(5): 474-8. | Prophylaxis for Caesarean section |
| 1. Pitt C, Sanchez-Ramos L, Kaunitz AM. Adjunctive intravaginal metronidazole for the prevention of postcesarean endometritis: A randomized controlled trial. Obstet Gynecol 2001; 98(5): 745-50. | Prophylaxis for Caesarean section |
| 1. Polk BF, Schoenbaum SC. Prophylactic antibiotics in obstetrics. Clin Obstet Gynecol 1979; 22(2): 379-84. | Review |
| 1. Pothinam S, Chanpoo T, Lumbiganon P. Post-cesarean section puerperal morbidity. The incidence and risk factors at Srinagarind Hospital. J Med Assoc Thai 1992; 75(3): 173-7. | Prophylaxis for Caesarean section |
| 1. Poulain P, Betremieux P, Donnio PY, Proudhon JF, Karege G, Giraud JR. Selective intrapartum anti-bioprophylaxy of group B streptococci infection of neonates: A prospective study in 2454 subsequent deliveries. Eur J Obstet Gynecol Reprod Biol 1997; 72(2): 137-40. | >10% had symptoms in labour |
| 1. Puopolo KM, Madoff LC, Eichenwald EC. Early-onset group B streptococcal disease in the era of maternal screening. Pediatrics 2005; 115(5): 1240-6. | No data on adverse events |
| 1. Rayburn W, Varner M, Galask R. Comparison of moxalactam and cefazolin as prophylactic antibiotics during cesarean section. Antimicrob Agents Chemother 1985; 27(3): 337-9. | Prophylaxis for Caesarean section |
| 1. Raymond J, Lopez E, Bonacorsi S, et al. Evidence for transmission of escherichia coli from mother to child in late-onset neonatal infection. Pediatr Infect Dis J 2008; 27(2): 186-8. | Case report |
| 1. Reggiori A, Ravera M, Cocozza E, Andreata M, Mukasa F. Randomized study of antibiotic prophylaxis for general and gynaecological surgery from a single centre in rural Africa. The British journal of surgery, 1996. | Surgical prophylaxis |
| 1. Rehu M, Jahkola M. Prophylactic antibiotics in Caesarean section: effect of a short preoperative course of benzyl penicillin or clindamycin plus gentamicin on postoperative infectious morbidity. Ann Clin Res 1980; 12(2): 45-8. | Prophylaxis for Caesarean section |
| 1. Renner RM, Renner A, Schmid S, et al. Efficacy of a strategy to prevent neonatal early-onset group B streptococcal (GBS) sepsis. J Perinat Med 2006; 34(1): 32-8. | No data on adverse events |
| 1. Rentz AC, Samore MH, Stoddard GJ, Faix RG, Byington CL. Risk factors associated with ampicillin-resistant infection in newborns in the era of group B streptococcal prophylaxis. Arch Pediatr Adolesc Med 2004; 158(6): 556-60. | >10% symptomatic (chorioamnionitis and prolonged rupture of membranes) |
| 1. Rijhsinghani A, Savopoulos SE, Walters JK, Huggins G, Hibbs JR. Ampicillin/sulbactam versus ampicillin alone for cesarean section prophylaxis: A randomized double-blind trial. Am J Perinatol 1995; 12(5): 322-4. | Prophylaxis for Caesarean section |
| 1. Roex AJ, Van Loenen AC. Pharmacokinetics of three-dose cefoxitin prophylaxis in caesarean section. Pharm Weekbl Sci 1988; 10(6): 281-3. | Prophylaxis for Caesarean section |
| 1. Roex AJM, Puyenbroek JI, Van Loenen AC, Arts NFT. Single- versus three-dose cefoxitin prophylaxis in caesarean section: A randomized clinical trial. Eur J Obstet Gynecol Reprod Biol 1987; 25(4): 293-8. | Prophylaxis for Caesarean section |
| 1. Roth P, Schaal JP, Fromentin C, Guerrier T, Maillet R, Colette C. [Comparative study of 2 protocols for antibiotic therapy. Maternal-fetal non-specific bacterial infections during labor]. Journal de gynécologie, obstétrique et biologie de la reproduction, 1990. | Full text not in English |
| 1. Rothbard MJ, Mayer W, Wystepek A, Gordon M. Prophylactic antibiotics in cesarean section. Obstet Gynecol 1975; 45(4): 421-4. | Prophylaxis for Caesarean section |
| 1. Rouse DJ, Hauth JC, Andrews WW, Mills BB, Maher JE. Chlorhexidine vaginal irrigation for the prevention of peripartal infection: A placebo-controlled randomized clinical trial. Am J Obstet Gynecol 1997; 176(3): 617-22. | Not systemic prophylaxis |
| 1. Rudge MV, Atallah AN, Peracoli JC, Tristao Ada R, Mendonca Neto M. Randomized controlled trial on prevention of postcesarean infection using penicillin and cephalothin in Brazil. Acta Obstet Gynecol Scand 2006; 85(8): 945-8. | Prophylaxis for Caesarean section |
| 1. Saad A, Finan R, Papas S, Anastabiades E. Evaluation of ceftizoxime in the prophylaxis of gynecological surgery. Revue Medicale Libanaise 2004; 16(1): 36-8. | Surgical prophylaxis and timing of antibiotics also unclear. |
| 1. Sabir S. Infective morbidity following Caesarean section. Specialist 1996; 13(1): 29-32. | Prophylaxis for Caesarean section |
| 1. Saezllorens X, Ahchu MS, Castano E, et al. Intrapartum Prophylaxis with Ceftriaxone Decreases Rates of Bacterial-Colonization and Early-Onset Infection in Newborns. Clin Infect Dis 1995; 21(4): 876-80. | >10% symptomatic (prolonged rupture of membranes) |
| 1. Saltzman DH, Eron LJ, Tuomala RE, Protomastro LJ, Sites JG. Single-dose antibiotic prophylaxis in high-risk patients undergoing cesarean section. A comparative trial. J Reprod Med 1986; 31(8): 709-12. | Prophylaxis for Caesarean section |
| 1. Schrag SJ, Cutland CL, Zell ER, et al. Risk factors for neonatal sepsis and perinatal death among infants enrolled in the prevention of perinatal sepsis trial, Soweto, South Africa. Pediatr Infect Dis J 2012; 31(8): 821-6. | >10% symptomatic (prolonged rupture of membrane, foul smelling vaginal discharge) |
| 1. Schrag SJ, Hadler JL, Arnold KE, Martell-Cleary P, Reingold A, Schuchat A. Risk factors for invasive, early-onset Escherichia coli infections in the era of widespread intrapartum antibiotic use. Pediatrics 2006; 118(2): 570-6. | >10% symptomatic (intrapartum fever, prolonged rupture of membrane) |
| 1. Schuchat A, Zywicki SS, Dinsmoor MJ, et al. Risk factors and opportunities for prevention of early-onset neonatal sepsis: A multicenter case-control study. Pediatrics 2000; 105(1): 21-6. | >10% symptomatic (intrapartum fever, prolonged rupture of membrane) |
| 1. Sengupta A, Kohli JK. Antibiotic prophylaxis in cesarean section causing anaphylaxis and intrauterine fetal death. J Obstet Gynaecol Res 2008; 34(2): 252-4. | Case report |
| 1. Shrestha B, Marhatha R, Giri A, Jaisi S, Maskey U. Surgical site wound infection in relation to antibiotic prophylaxis given before skin incision and after cord clamping during cesarean delivery. Nepal Med Coll J 2014; 16(2-4): 148-51. | Prophylaxis for Caesarean section |
| 1. Simchen E, Shapiro M, Michel J, Sacks TG. The successful use of antibiotic prophylaxis in selected high-risk surgical patients under non-trial, everyday conditions. J Hosp Infect 1980; 1(3): 211-20. | Surgical prophylaxis |
| 1. Singleton ML. Group B strep prophylaxis: what are we creating? Midwifery Today Int Midwife 2007; (81): 18-20. | Editorial |
| 1. Skjeldestad FE, Bjornholt JV, Gran JM, Erisken HM. The effect of antibiotic prophylaxis guidelines on surgical-site infections associated with cesarean delivery. International Journal of Gynecology and Obstetrics 2014; 128(2): 126-30. | Prophylaxis for Caesarean section |
| 1. Smith AM, Cox CWFM. Necrotising fasciitis following caesarean section. Journal of Obstetrics and Gynaecology 1992; 12(4): 246-7. | Case report |
| 1. Spandorfer SD, Graham E, Forouzan I. Postcesarean endometritis. Clinical risk factors predictive of positive blood cultures. J Reprod Med 1996; 41(11): 797-800. | Prophylaxis for Caesarean section |
| 1. Spreafico P, Scian A, Epis A, Vassen L, Bonazzi C, Lovotti M. Cesarean section: antibiotic prophylaxis with ceftezole. Chemioterapia 1987; 6(2 Suppl): 613-6. | Prophylaxis for Caesarean section |
| 1. Stage AH, Glover DD, Vaughan JE. Low-dose cephradine prophylaxis in obstetric and gynecologic surgery. J Reprod Med 1982; 27(3): 113-9. | Surgical prophylaxis |
| 1. Stark MA, Ross MF, Kershner W, Searing K. Case Study of Intrapartum Antibiotic Prophylaxis and Subsequent Postpartum Beta-Lactam Anaphylaxis. Jognn 2015; 44(5): 610-7. | Case report |
| 1. Stiver HG, Forward KR, Livingstone RA. Double blind placebo-controlled multicentre comparison of cefoxitin vs cefazolin prophylaxis against post-cesarean section infection. Clinical and Investigative Medicine 1982; 5(2-3): 34B. | Abstract |
| 1. Stiver HG, Forward KR, Livingstone RA. Multicenter comparison of cefoxitin versus cefazolin for prevention of infectious morbidity after nonelective cesarean section. Am J Obstet Gynecol 1983; 145(2): 158-63. | Prophylaxis for Caesarean section |
| 1. Stiver HG, Forward KR, Tyrrell DL, et al. Comparative cervical microflora shifts after cefoxitin or cefazolin prophylaxis against infection following cesarean section. Am J Obstet Gynecol 1984; 149(7): 718-21. | Prophylaxis for Caesarean section |
| 1. Sullivan SA, Smith T, Chang E, Hulsey T, Vandorsten JP, Soper D. Administration of cefazolin prior to skin incision is superior to cefazolin at cord clamping in preventing postcesarean infectious morbidity: a randomized, controlled trial.[Erratum appears in Am J Obstet Gynecol. 2007 Sep;197(3):333]. Am J Obstet Gynecol 2007; 196(5): 455.e1-5. | Prophylaxis for Caesarean section |
| 1. Suonio S, Saarikoski S, Vohlonen I, Kauhanen O. Risk factors for fever, endometritis and wound infection after abdominal delivery. Int J Gynaecol Obstet 1989; 29(2): 135-42. | Prophylaxis for Caesarean section |
| 1. Szalontay AS. [Antibiotic prophylaxis in cesarean section]. Revista medico-chirurgicală̆ a Societă̆ţ̜ii de Medici ş̧i Naturaliş̧ti din Iaş̧i, 1997. | Full text not in English |
| 1. Tassi PG, Tarantini M, Rampinelli F, et al. Piperacillin in antibiotic prophylaxis: a single-dose administration for cesarean section. J Chemother 1989; 1(4 Suppl): 1025-6. | Prophylaxis for Caesarean section |
| 1. Tassi PG, Tarantini M, Cadenelli GP, Gastaldi A, Benedetti M. Ceftazidime in antibiotic prophylaxis for emergency cesarean section: a randomized prospective study. Int J Clin Pharmacol Ther Toxicol 1987; 25(10): 582-8. | Prophylaxis for Caesarean section |
| 1. Teo SM, Mok D, Pham K, et al. The Infant Nasopharyngeal Microbiome Impacts Severity of Lower Respiratory Infection and Risk of Asthma Development. Cell Host Microbe 2015; 17(5): 704-15. | Unclear when antibiotics were given and delivery mode |
| 1. Thigpen BD, Hood WA, Chauhan S, et al. Timing of prophylactic antibiotic administration in the uninfected laboring gravida: a randomized clinical trial. Am J Obstet Gynecol, 2005. | Prophylaxis for Caesarean section |
| 1. Thurman AR, Anca Y, White CA, Soper DE. Post-cesarean delivery infectious morbidity: Focus on preoperative antibiotics and methicillin-resistant Staphylococcus aureus. Am J Infect Control 2010; 38(8): 612-6. | Prophylaxis for Caesarean section |
| 1. To WW, Lau WN. A protocol of selective antibiotic prophylaxis for caesarean section based on risk factors. Aust N Z J Obstet Gynaecol 2001; 41(4): 402-6. | Prophylaxis for Caesarean section |
| 1. Towers CV, Cart MH, Padilla G, Asrat T. Potential consequences of widespread antepartal use of ampicillin. Am J Obstet Gynecol 1998; 179(4): 879-83. | Unable to distinguish mothers treated in labour from mother treated in pregnancy as well |
| 1. Tsai CH, Chen YY, Wang KG, Chen CY, Chen CP. Characteristics of early-onset neonatal sepsis caused by Escherichia coli. Taiwan J Obstet Gynecol 2012; 51(1): 26-30. | Unable to distinguish women who had emergency caesarean section from those that had elective caesarean section |
| 1. Tully JL, Klapholz H, Baldini LM, Friedland GH. Perioperative use of cefoxitin in primary cesarean section. J Reprod Med 1983; 28(12): 827-32. | Prophylaxis for Caesarean section |
| 1. Tuppurainen N, Hallman M. Prevention of neonatal group B streptococcal disease: intrapartum detection and chemoprophylaxis of heavily colonized parturients. Obstet Gynecol, 1989. | No data on adverse events |
| 1. Turner MJ. Prophylactic antibiotics for caesarean section and hysterectomy. Journal of Obstetrics and Gynaecology 1994; 14(1): 54-5. | Editorial |
| 1. Tzingounis V, Makris N, Zolotas J. Cefuroxime prophylaxis in caesarean section. Pharmatherapeutica 1982; 3(2): 140-2. | Prophylaxis for Caesarean section |
| 1. van der Linden MC, van Erp EJ, Ruijs GJ, Holm JP. A prospective randomized study comparing amoxycillin/clavulanate with cefuroxime plus metronidazole for perioperative prophylaxis in gynaecological surgery. Eur J Obstet Gynecol Reprod Biol 1993; 50(2): 141-5. | Surgical prophylaxis |
| 1. Van Scoy RE. Prophylactic antibiotic therapy: its use and abuse. Clin Obstet Gynecol 1976; 19(3): 721-33. | Review |
| 1. Varner MW, Weiner CP, Petzold CR, Galask RP. Comparison of cefotetan and cefoxitin as prophylaxis in cesarean section. Am J Obstet Gynecol, 1986. | Prophylaxis for Caesarean section |
| 1. von Mandach U, Huch R, Malinverni R, Huch A. Ceftriaxone (single dose) versus cefoxitin (multiple doses): success and failure of antibiotic prophylaxis in 1052 cesarean sections. J Perinat Med 1993; 21(5): 385-97. | Prophylaxis for Caesarean section |
| 1. Wali A, Taj Z, Abbas Z. Chemoprophylaxis in caesarean sections. Journal of the College of Physicians and Surgeons Pakistan 2002; 12(2): 78-81. | Prophylaxis for Caesarean section |
| 1. Wallace RL, Yonekura ML. The use of prophylactic antibiotics in patients undergoing emergency primary cesarean section. Am J Obstet Gynecol 1983; 147(5): 533-6. | Prophylaxis for Caesarean section |
| 1. Wax JR, Hersey K, Philput C, et al. Single dose cefazolin prophylaxis for postcesarean infections: before vs. after cord clamping. J Matern Fetal Med 1997; 6(1): 61-5. | Prophylaxis for Caesarean section |
| 1. Wegienka G, Havstad S, Zoratti EM, Kim H, Ownby DR, Johnson CC. Combined effects of prenatal medication use and delivery type are associated with eczema at age 2 years. Clin Exp Allergy 2015; 45(3): 660-8 | Timing of antibiotics unclear and unable to distinguish between antibiotics and antifungals |
| 1. Weinberg M, Fuentes JM, Ruiz AI, et al. Reducing infections among women undergoing cesarean section in Colombia by means of continuous quality improvement methods. Arch Intern Med 2001; 161(19): 2357-65. | >10% participants had elective caesarean section |
| 1. Weissberg SM, Edwards NL, O'Leary JA. Prophylactic antibiotics in cesarean section. Obstet Gynecol 1971; 38(2): 290-3. | Prophylaxis for Caesarean section |
| 1. Westen EHMN, Kolk PR, Van Velzen CL, et al. Single-dose compared with multiple day antibiotic prophylaxis for cesarean section in low-resource settings, a randomized controlled, noninferiority trial. Acta Obstet Gynecol Scand 2015; 94(1): 43-9. | Prophylaxis for Caesarean section |
| 1. Wolfe HM, Gross TL, Sokol RJ, Bottoms SF, Thompson KL. Determinants of morbidity in obese women delivered by cesarean. Obstet Gynecol 1988; 71(5): 691-6. | Prophylaxis for Caesarean section |
| 1. Wong R, Gee CL, Ledger WJ. Prophylactic use of cefazolin in monitored obstetric patients undergoing cesarean section. Obstet Gynecol 1978; 51(4): 407-11. | Prophylaxis for Caesarean section |
| 1. Work BA, Jr. Role of preventive antibiotics in patients undergoing cesarean section. South Med J 1977; 70 Suppl 1: 44-5. | Prophylaxis for Caesarean section |
| 1. Yip SK, Lau TK, Rogers MS. A study on prophylactic antibiotics in cesarean sections - Is it worthwhile? Acta Obstet Gynecol Scand 1997; 76(6): 547-9. | Prophylaxis for Caesarean section |
| 1. Yonekura ML, Appleman M, Wallace R, Boucher M, Nakamura R. Predictive value of amniotic-membrane cultures for the development of postcesarean endometritis. Rev Infect Dis 1984; 6 Suppl 1: S157-64. | Prophylaxis for Caesarean section |
| 1. Young BC, Hacker MR, Dodge LE, Golen TH. Timing of antibiotic administration and infectious morbidity following cesarean delivery: incorporating policy change into workflow. Arch Gynecol Obstet 2012; 285(5): 1219-24. | Prophylaxis for Caesarean section |
| 1. Young R, Platt L, Ledger W. Prophylactic cefoxitin in cesarean section. Surg Gynecol Obstet 1983; 157(1): 11-4. | Prophylaxis for Caesarean section |
| 1. Zhang J, Johnson CD, Hoffman M. Cervical cerclage in delayed interval delivery in a multifetal pregnancy: a review of seven case series. Eur J Obstet Gynecol Reprod Biol 2003; 108(2): 126-30. | Review of case series about cervical cerclage for multiple births |

**Table 1: Characteristics and findings of individual studies**

| **Study,**  **Country** | **Study design** | | **Participants** | **Treatment** | **Outcome** | **Results**  **Treatment Control** | | **Summary measure (95%CI)** | **Covariates adjusted for in analysis** |
| --- | --- | --- | --- | --- | --- | --- | --- | --- | --- |
| **Outcomes used to investigate the adverse events of IAP** | | | | | | | | | |
| Aloisio 2014^1^  Italy | Cohort | | 52 infants  6-7 days old  (26 treated,  26 not treated) | GBS prophylaxis  Intrapartum 2g ampicillin at least 4h before delivery, followed by 1g every 4h until delivery | Gut microbiota:  *E. coli*  *Bacteroides fragilis*  *Bifidobacterium spp.*  *Clostridium difficile*  *Lactobacillus* spp. | M: 8.18  (R: 4.09-12.70)  M: 8.17  (R: 4.68-11.99)  M: 5.85  (R: 3.24–7.79)  M: 3.89  (R: 3.12–4.80)  M: 6.69  (R: 5.40–8.93) | M: 9.03  (R: 5.61-11.78)  M: 8.53  (R: 5.22-11.16)  M: 7.29  (R: 4.12–10.95)  M: 3.70  (R: 2.85–5.46)  M: 6.73  (R: 5.45–8.20) | NS  NS  p = 0.001  NS  NS | None |
| Aloisio 2016^2^  Italy | Prospective cohort | | 20 infants  6-7 days old  (10 treated, 10 not treated) | GBS prophylaxis  Intrapartum 2g ampicillin at least 4 h before delivery, followed by 1 g every 4 h until delivery | Gut microbiota composition:  *Actinobacteria*  *Bacteriodetes*  *Proteobacteria Firmicutes* | 0.4%  16.0%  54.7%  - | 3.8%  47.7%  15.5%  - | p < 0.05  p < 0.05  p < 0.05  - | None  None  None  - |
|  |  |  |  |  | Gut microbiota composition:  *Bifidobacteriaceae* genus  Other microbial genus | 0.02% | 6.469% | p < 0.05  non-significant | None |
|  |  |  |  |  | Gut microbiota: Sample richness and biodiversity | Alpha diversity  The control group showed a more complex microbial profile compared to the IAP group who had a reduced level of richness and biodiversity: Chao1 and Shannon indexes: p = 0.0081 and p = 0.036, respectively; Bray-Curtis index: p > 0.05.  Beta diversity  Significant phylogenetic and relative abundance difference: unweighted UniFrac distance, p < 0.05. Principal coordinate analysis based on Weighted and Unweighted UniFrac distances at genus level shows segregation along axis1 for both UniFrac indices, indicating a separation in two clusters due to IAP treatment.  Diversity at bacterial family level  A more complex profile in control compared to IAP group in terms of biodiversity, with a more equal distribution at family/genus level. The IAP group had a lower number of bacterial families with some cases composed almost exclusively by *Enterobacteriaceae* family members (*Proteobacteria*) that can reach over 90% of relative abundance and a few samples are even characterised by the presence of *Streptococcaceae* family (with an average of 13%). | | | None |
| Arboleya 2015^3^  Spain | Cohort | | 27 preterm infants  2-90 days old (14 treated, 13 not treated) | Indication unknown  1 mother received a single dose of penicillin, and 1 mother received 1 dose of ampicillin every 6 hours for 3 days. 12 mothers received ampicillin plus erythromycin (between 2 and 24 doses of each antibiotic) | Gut microbiota composition | Cluster analysis  Day 2: Higher percentage of sequences from *Leuconostaceae* in controls.  Day 10: Higher percentage of sequences from *Micrococcaceae* and *Propionibacteriaceae* in controls.  Day 30: Higher relative amounts of *Comamonadaceae, Staphylococcaceae*, and unclassified *Bacilli* in controls.  Higher *Bifidobacteriaceae*, *Streptococcaceae,* unclassified *Actinobacteria*, and unclassified *Lactobacillales* (p < 0.05) in controls.  Lower percentage of *Enterobacteriaceae* in controls (p < 0.05)  Day 90: Most differences disappeared except in *Ruminococcaceae* microbial group (differences unclear)  Quantitative PCR:  Day 2 and 10: No significant differences  Day 30: Higher amounts of *Staphylococcaceae* in control.  Lower amounts of *Enterobacteriaceae* and total bacteria in control.  Day 90: higher amounts of *bifidobacteria* in control. | | | None |
| Arboleya 2016^4^  Spain | Cohort (same cohort as above Arboleya 2015 study) | | 27 preterm infants  2-90 days old (14 treated, 13 not treated) (same cohort as above) | Indication unknown  1 mother received a single dose of penicillin, and 1 mother received 1 dose of ampicillin every 6 hours for 3 days. 12 mothers received ampicillin plus erythromycin (between 2 and 24 doses of each antibiotic) | Gut microbiota composition | Day 1: no statistically significant differences on the bacterial phyla  Day 30: higher relative frequency of *Actinobacteria* phylum (p < 0.05) and *Firmicutes* phylum (p < 0.01) in controls. Lower frequency of *Proteobacteria* phylum in controls. Higher levels of acetic (p = 0.075) and total (p = 0.060) short chain fatty acids in controls. | | | None |
| Ashkenazi-Hoffnung 2011^5^  Israel | Case-control | | 195 infants  7-90 days old  (17 treated, 178 not treated) | GBS prophylaxis  94% ampicillin | Late-onset serious bacterial infections | 8 | 63 | OR per dose of IAP:  5.19 (0.01-93.11) | Infant age, maternal age  birth weight, gestational age, type of delivery, and GBS status had no significant effect.  Number of doses, time from antibiotic administration to delivery |
|  |  |  |  |  | Ampicillin resistant late-onset serious bacterial infections | 85% = 14.45/17 (Note: Numbers do not add up - 14 people would be 82% and 15 people would be 88%) | 63% = 112/178 | p = 0.19 | None – “Multivariate logistic regression did not identify any variable that was significantly associated with increased risk of resistance to ampicillin or FGCs” |
|  |  |  |  |  | First-generation cephalosporin resistant late-onset serious bacterial infections | 57% = 9.69/17  (Note: Numbers do not add up - 9 people would be 53% and 10 people would be 59%) | 26% = 46/178 | p = 0.19 | None – “Multivariate logistic regression did not identify any variable that was significantly associated with increased risk of resistance to ampicillin or FGCs” |
|  |  |  |  |  | First-generation cephalosporin resistance in UTI only | 75% (unable to calculate numbers) | 23.5% (unable to calculate numbers) | p = 0.04 | None |
|  |  |  |  |  | Ampicillin resistant *E. coli* only | 100% (unable to calculate numbers) | 54.5% (unable to calculate numbers) | p = 0.14 | None |
|  |  |  |  |  | First-generation cephalosporin resistant *E. coli* only | 60% (unable to calculate numbers) | 22.7% (unable to calculate numbers) | p = 0.21 | None |
|  |  |  |  |  | Gentamicin or third generation cephalosporin resistance | 0 | 0 | - | - |
| Balter 2003^6^  USA | Retrospective cohort | | 261 infants  (81 treated, 180 not treated) | GBS prophylaxis (59%)  Other reasons (39%)  Maternal fever (6%)  Antibiotic not reported | 5 minute APGAR score | Median: 8  IQR: 8-9 | Median: 8  IQR: 8-9 | - | None |
|  |  |  |  |  | Complete blood count | 21 | 17 | *RR: 2.75 (1.53–4.92)* | None |
|  |  |  |  |  | Blood culture drawn | 10 | 10 | *RR: 2.22 (0.96–5.13)* | None |
|  |  |  |  |  | Urine culture via catheterisation | 2 | 1 | *RR: 4.44 (0.41-48.32)* | None |
|  |  |  |  |  | Any urine culture | 4 | 2 | *RR: 4.44 (0.83-23.78)* | None |
|  |  |  |  |  | Chest radiograph | 3 | 8 | *RR: 0.83 (0.23-3.06)* | None |
|  |  |  |  |  | Infant given antibiotics within 7 days | 6 | 8 | *RR: 1.67 (0.60-4.65)* | None |
|  |  |  |  |  | Infant given intravenous catheter | 4 | 8 | *RR: 1.11 (0.34-3.58)* | None |
|  |  |  |  |  | Infant in NICU | 3 | 7 | *RR: 0.95 (0.25-3.59)* | None |
|  |  |  |  |  | Mechanical ventilation | 1 | 0 | - | None |
|  |  |  |  |  | Supplemental oxygen | 5 | 9 | *RR: 1.23 (0.43-3.57)* | None |
|  |  |  |  |  | Hospitalisation ≥ 48 hours | 14 | 12 | *RR: 2.59 (1.26-5.35)* | None |
|  |  |  |  |  | Hospitalisation > 72 hours | 14 | 17 | *RR: 1.83 (0.95-3.53)* | None |
|  |  |  |  |  | Length of hospitalisation | Median:  56.8 hours | Median:  47 hours | p = 0.02 | None |
| Briody 2016^7^  USA | Retrospective cohort | | 165 intrapartum women  (73 received ‘appropriate’ IAP, 92 received ‘inappropriate’ IAP) | GBS prophylaxis  Appropriate IAP: Penicillin, Cefazolin  Inappropriate IAP: Clindamycin, Erythromycin, Vancomycin | Neonate placed on antibiotics | 3 | 4 | *RR: 0.94 (0.22-4.09)* | None |
|  |  |  |  |  | Hospital stay > 2 days | 25 | 22 | *RR: 1.43 (0.88-2.32)* | None |
|  |  |  |  |  | Hospital stay > 3 days | 15 | 16 | *RR: 1.18 (0.63-2.23)* | None |
|  |  |  |  |  | 5 minute APGAR score | M: 9 (R: 5-10) | M: 9 (R: 3-10) | p = 0.24 | None |
|  |  |  |  |  | Number of blood cultures performed | M: 2 (SD: 2.7) | M: 9 (SD: 9.9) | p = 0.11 | None |
| Corvaglia 2016 ^8^  Italy | Prospective cohort | | 84 infants  7-30 days old  (35 treated, 49 not treated) | GBS Prophylaxis  Intravenous ampicillin every 4 hours until delivery (first dose 2 g, following doses 1 g each) | Gut Microbiota composition: *Bifidobacterium spp.*  7 days  *Bifidobacterium spp.* 30 days  *Lactobacillus spp.*  7 days  *Lactobacillus spp.*  30 days  *Bacteroides fragilis* *spp.*  7 days  *Bacteroides fragilis spp.*  30 days | Median: 6.01 (IQR: 5.51–6.98)  Median: 8.41 (IQR: 7.71-8.80)  Median: 5.56 (IQR: 4.94-6.14)  Median: 5.29 (IQR: 4.68-6.01)  Median: 7.71 (IQR: 5.80-9.33)  Median: 7.36  (IQR 5.80-9.09) | Median: 7.80 (IQR: 6.61–8.26)  Median: 8.39 (IQR: 7.96-8.86)  Median: 5.45 (IQR: 4.81-6.14)  Median: 5.25 (IQR: 4.60-6.15)  Median: 7.75 (IQR: 5.87-9.61)  Median: 8.51 (IQR: 5.86-9.37) | p = 0.000  p = 0.363  p = 0.872  p = 0.932  p > 0.05  p > 0.05 | Feeding |
| Cox 1996^9^  USA | Randomised controlled trial | | 78 intrapartum women  (39 treated, 39 not treated) | Preterm labour  2g ampicillin and 1g sulbactam parenterally every 6 hours for 8 doses, followed by ampicillin-clavunate 250mg orally every 8 hours for 5 days. | Symptomatic vulvovaginitis caused by *Candida albicans* | 27 | Not stated | - | - |
|  |  |  |  |  | Pseudo-membranous enterocolitis caused by *Clostridium difficile* | 1 | Not stated | - | - |
| Dinsmoor 2005^10^  USA | Retrospective cohort | | 435 mother-infant pairs 0-1 month post-partum  (173 treated, 262 not treated) | 136 for GBS Prophylaxis. Other mothers received antibiotics for other indications | Neonatal thrush | 21 | 18 | OR: 1.87 (0.97-3.63) | None |
|  |  |  |  |  | Maternal thrush | 22 | 17 | OR: 2.1 (1.08-4.08) | None |
|  |  |  |  |  | Total candidiasis | 26 | 20 | OR: 2.14 (1.15-3.97) | None |
| Glasgow 2005^11^  USA | Case-control | | 182 infants  7-90 days old (62 treated, 120 not treated) | Indication unknown  Penicillin, ampicillin, or broad spectrum | Late-onset serious bacterial infection | 37  Penicillin only: 10/23  Broad-spectrum: 29/39 | 53  80  61 | OR: 1.96 (1.05–3.66)  OR: 0.95 (0.37-2.44)  OR: 4.95 (2.04–11.98) | Hospital of delivery, maternal chorioamnionitis and breastfeeding |
|  |  |  |  |  | Ampicillin-resistant late-onset serious bacterial infections | 24  Penicillin only: 4/9  Ampicillin only: 12/18  Other IAP: 8/10 | 13  33  25  29 | OR: 5.7 (2.3–14.3)  OR: 2.5 (0.6-10.6)  OR: 6.2 (1.9-19.7)  OR: 12.3 (2.3-65.5) | Hospital of delivery |
|  |  |  |  |  | Ampicillin resistant UTI infections | Not reported | Not reported | OR: 4.3 (1.6–11.7) | Hospital of delivery |
|  |  |  |  |  | Other serious bacterial infections (meningitis, omphalitis, and bacteraemia without UTI) | Not reported | Not reported | OR: 25 (1.8–346) | Hospital of delivery |
| Gordon 1995^12^  USA | Randomised controlled trial | | 117 intrapartum women  (58 treated, 59 not treated) | Preterm labour  Ceftizoxime for 5 days or 3 days | Bleeding abnormalities | 0 | - | - | - |
|  |  |  |  |  | *Clostridium difficile* colitis | 0 | - | - | - |
|  |  |  |  |  | Multi-resistant bacterial infections | 0 | - | - | - |
| Jaureguy 2004^13^  France | Prospective cohort | | 50 infants  3 days old  (25 treated, 25 not treated) | GBS Prophylaxis  Intravenous 2g amoxicillin at the time of labour and then 1g every 4 h until delivery | Gut microbiota:  Numbers colonised with:  *Enterobacteria*  *Enterococci*  *Staphylococci*  *Bacteroides*  *Clostridium*  *Bifidobacterium* | 13  15  21  13  3  6 | 16  17  22  7  10  12 | p = 0.58  p = 0.73  p = 1.00  p = 0.15  p = 0.04  p = 0.18 | None |
|  |  |  |  |  | Gut microbiota composition (log CFU/gram):  *Enterobacteria*  *Enterococci*  *Staphylococci*  *Bacteroides*  *Clostridium*  *Bifidobacterium* | Median: 8.4  (R: 3.3–9.5)  Median 8.3  (R: 3.6–10.3)  Median: 6.5  (R: 3.6–8.0)  Median: 8.0  (R: 6.3–10.3)  Median: 5.3  (R: 4.3–5.8)  Median: 8.2  (R: 4.3–9.5) | Median: 9.2  (R: 3.3–9.8)  Median: 7.3  (R: 3.3–9.5)  Median: 7.0  (R: 4.0–9.3)  Median: 7.9  (R: 3.6–9.6)  Median: 6.2  (R: 3.6–8.1)  Median: 8.5  (R: 6.9-10.3) | p = 0.18  p = 0.78  p = 0.53  p = 0.12  p = 0.01  p = 0.10 | None |
|  |  |  |  |  | Amoxicillin-resistant *Enterobacteria* | 10 | 12 | *RR: 0.83 (0.44-1.56)* | None |
|  |  |  |  |  | Amoxicillin-resistant *E. coli* | 6 | 11 | *RR: 0.55 (0.24-1.25)* | None |
| Kampikaho 1993^14^  Uganda | Quasi-randomised controlled trial | | 660 intrapartum women  (330 treated, 330 not treated) | Post-partum infection prevention  1g streptomycin or 0.8MU penicillin | Side effects | 0 | - | - | - |
| Keettel 1949^15^  USA | Controlled trial | | 895 intrapartum women  (465 treated, 430 not treated) | Post-partum infection prevention  300,000/600,000 units of penicillin at the indication of labour and then after 24-hour intervals. | Mild urticaria | 7 | - | - | - |
|  |  |  |  |  | General urticaria | 2 (8-12 days, 600,000 units) | - | - | - |
|  |  |  |  |  | Local allergic manifestations | 5 (900,000 units) | - | - | - |
|  |  |  |  |  | Abscess formations at site of injections | 0 | - | - | - |
|  |  |  |  |  | Discomfort following injections | Relatively uncommon and never severe or persistent | - | - | - |
| Keettel 1950^16^  USA | Controlled trial | | 773 intrapartum women  (382 treated, 391 not treated) | Post-partum infection prevention  600,000 units of penicillin at the indication of labour, and then after 24-hour intervals. | General urticaria | 1 (8 days) | - | - | - |
|  |  |  |  |  | Local allergic manifestations | 1 | - | - | - |
|  |  |  |  |  | Abscess formations at the site of injections | 0 | - | - | - |
| Kenyon 2008^17^  UK | Factorial randomised trial | | 3173 children  0-7 years old (numbers differ for outcomes – see treatment column) | Spontaneous preterm labour  375 mg amoxicillin–clavulanate (n=763), 250 mg erythromycin (n=785), amoxicillin–clavulanate and erythromycin (n=796), double placebo (n=735) | Mild functional impairment | ERY and AMC: 181/769  ERY: 191/785  AMC: 168/763 | 151/735 | OR: 1.00 (reference)  OR: 1.24 (0.96–1.60)  OR: 1.29 (1.00–1.65)  OR: 1.10 (0.85–1.42) | Maternal baseline, social class, and other factors |
|  |  |  |  |  | Moderate functional impairment | ERY and AMC: 91/769  ERY: 94/785  AMC: 85/763 | 77/735 | OR: 1.00 (reference)  OR: 1.22 (0.88–1.70)  OR: 1.24 (0.89–1.72)  OR: 1.09 (0.78–1.53) | Maternal baseline, social class, and other factors |
|  |  |  |  |  | Severe functional impairment | ERY and AMC: 53/769  ERY: 48/785  AMC: 46/763 | 47/735 | OR: 1.00 (reference)  OR: 1.17 (0.77–1.77)  OR: 1.04 (0.68–1.59)  OR: 0.97 (0.63–1.49) | Maternal baseline, social class, and other factors |
|  |  |  |  |  | Any functional impairment | ERY and AMC: 325/769  ERY: 333/785  AMC: 299/763 | 275/735 | OR: 1.00 (reference)  OR: 1.22 (1.00–1.51)  OR: 1.23 (1.00–1.51)  OR: 1.08 (0.88–1.33) | Maternal baseline, social class, and other factors |
|  |  |  |  |  | Three or more abnormal attributes | ERY and AMC: 72/769  ERY: 59/785  AMC: 75/763 | 74/735 | OR: 1.00 (reference)  OR: 0.92 (0.66–1.30)  OR: 0.73 (0.51–1.04)  OR: 0.97 (0.69–1.37) | Maternal baseline, social class, and other factors |
|  |  |  |  |  | Cerebral palsy | ERY and AMC: 35/769  ERY: 18/785  AMC: 15/763 | 12/735 | OR: 1.00 (reference)  OR: 2.91 (1.50–5.65)  OR: 1.42 (0.68–2.98)  OR: 1.22 (0.57–2.62) | Maternal baseline, social class, and other factors |
|  |  |  |  | Any erythromycin, 250mg (n=1554), no erythromycin (n=1498) | Functional impairment | None: 896  Mild: 372  Moderate: 185  Severe: 101  Any: 658  Three or more abnormal attributes: 131 | None: 924  Mild: 319  Moderate: 162  Severe: 93  Any: 574  Three or more abnormal attributes: 149 | OR: 1.00 (reference)  OR: 1.20 (1.01–1.43)  OR: 1.18 (0.94–1.48)  OR: 1.12 (0.83–1.51)  OR: 1.18 (1.02–1.37)  OR: 0.83 (0.65–1.07) | Maternal baseline, social class, and other factors |
|  |  |  |  |  | Behaviour | Emotional symptoms: 327  Conduct problems: 480  Hyperactivity: 424  Peer problems: 405  Prosocial behaviour: 122  Overall difficulties: 384  Impact on families: 334 | Emotional symptoms: 330  Conduct problems: 420  Hyperactivity: 415  Peer problems: 391  Prosocial behaviour: 99  Overall difficulties: 363  Impact on families: 292 | OR: 0.94 (0.79–1.12)  OR: 1.15 (0.98–1.34)  OR: 0.98 (0.84–1.15)  OR: 1.00 (0.85–1.17)  OR: 1.20 (0.91–1.59)  OR: 1.03 (0.87–1.21)  OR: 1.13 (0.95–1.35) | Maternal baseline, social class, and other factors |
|  |  |  |  | Any erythromycin, 250mg (n=1611), no erythromycin (n=1562) | Cerebral palsy | 53 | 27 | OR: 1.93 (1.21–3.09) | Maternal baseline, social class, and other factors |
|  |  |  |  |  | Seizures | 149 | 116 | OR: 1.27 (0.99–1.64) | Maternal baseline, social class, and other factors |
|  |  |  |  |  | Seizures on prescribed medication | 27 | 17 | OR: 1.55 (0.84–2.85) | Maternal baseline, social class, and other factors |
|  |  |  |  |  | Hydrocephalus with shunt | 2 | 3 | OR: 0.65 (0.11–3.87) | Maternal baseline, social class, and other factors |
|  |  |  |  |  | ADHD from SDQ or parental report | 120 | 116 | OR: 1.0 (0.77–1.31) | Maternal baseline, social class, and other factors |
|  |  |  |  |  | Other developmental problems | 10 | 15 | OR: 0.64 (0.29–1.44) | Maternal baseline, social class, and other factors |
|  |  |  |  |  | Wheezing in last year | 295 | 295 | OR: 0.96 (0.81–1.15) | Maternal baseline, social class, and other factors |
|  |  |  |  |  | Medication for chest problems in last year | 262 | 280 | OR: 0.89 (0.74–1.07) | Maternal baseline, social class, and other factors |
|  |  |  |  |  | Admission to hospital in last year | 243 | 202 | OR: 1.20 (0.98–1.46) | Maternal baseline, social class, and other factors |
|  |  |  |  |  | Admission for chest problems | 32 | 38 | OR: 0.81 (0.51–1.31) | Maternal baseline, social class, and other factors |
|  |  |  |  |  | Diabetes | 0 | 2 | - | - |
|  |  |  |  |  | All bowel disorders | 64 | 38 | OR: 1.66 (1.10–2.49) | Maternal baseline, social class, and other factors |
|  |  |  |  | Any erythromycin, 250mg (n=2375), no erythromycin (n=2279) | Stillbirths | 20 | 24 | OR: 0.80 (0.44-1.45) | Maternal baseline, social class, and other factors |
|  |  |  |  |  | Deaths in first year | 61 | 41 | OR: 1.44 (0.96–2.14) | Maternal baseline, social class, and other factors |
|  |  |  |  |  | Deaths after first year | 5 | 5 | OR: 0.97 (0.28–3.34) | Maternal baseline, social class, and other factors |
|  |  |  |  |  | Total deaths | 86 | 70 | OR: 1.19 (0.86–1.63) | Maternal baseline, social class, and other factors |
|  |  |  |  | Any erythromycin, 250mg (n=1641), no erythromycin (n=1598) | Educational attainment (children failing to achieve level 2 or higher in national curriculum tests) | Reading: 377  Writing: 413  Maths: 239 | Reading: 367  Writing: 413  Maths: 225 | OR: 1.0 (0.96–1.04)  OR: 1.0 (0.97–1.04)  OR: 0.99 (0.96–1.03) | Maternal baseline, social class, and other factors |
|  |  |  |  | Any amoxicillin–clavulanate, 375 mg (n=1532), no amoxicillin–clavulanate (n=1520) | Functional impairment | None: 908  Mild: 349  Moderate: 176  Severe: 99  Any: 624  Three or more abnormal attributes: 147 | None: 912  Mild: 342  Moderate: 171  Severe: 95  Any: 608  Three or more abnormal attributes: 133 | OR: 1.00 (reference)  OR: 1.02 (0.86–1.22)  OR: 1.03 (0.82–1.30)  OR: 1.05 (0.78–1.41)  OR: 1.03 (0.89–1.19)  OR: 1.11 (0.87–1.42) | Maternal baseline, social class, and other factors |
|  |  |  |  |  | Behaviour | Emotional symptoms: 341  Conduct problems: 454 Hyperactivity: 418  Peer problems: 396  Prosocial behaviour: 112  Overall difficulties: 385 Impact on families: 312 | Emotional symptoms: 316  Conduct problems: 446 Hyperactivity: 421  Peer problems: 400  Prosocial behaviour: 109 Overall difficulties: 362 Impact on families: 314 | OR: 1.09 (0.92–1.30)  OR: 1.01 (0.87–1.18)  OR: 0.98 (0.84–1.15)  OR: 0.98 (0.83–1.15)  OR: 1.02 (0.78–1.34)  OR: 1.07 (0.91–1.27)  OR: 0.98 (0.82–1.17) | Maternal baseline, social class, and other factors |
|  |  |  |  | Any amoxicillin–clavulanate, 375 mg (n=1587), no amoxicillin–clavulanate (n=1586) | Cerebral palsy | 50 | 30 | OR: 1.69 (1.07–2.67) | Maternal baseline, social class, and other factors |
|  |  |  |  |  | Seizures | 144 | 121 | OR: 1.21 (0.94–1.56) | Maternal baseline, social class, and other factors |
|  |  |  |  |  | Seizures on prescribed medication | 22 | 22 | OR: 1.0 (0.55–1.81) | Maternal baseline, social class, and other factors |
|  |  |  |  |  | Hydrocephalus with shunt | 4 | 1 | OR: 4.01 (0.45–35.87) | Maternal baseline, social class, and other factors |
|  |  |  |  |  | ADHD from SDQ or parental report | 128 | 108 | OR: 1.20 (0.92–1.57) | Maternal baseline, social class, and other factors |
|  |  |  |  |  | Other developmental problems | 8 | 17 | OR: 0.47 (0.20–1.09) | Maternal baseline, social class, and other factors |
|  |  |  |  |  | Wheezing in last year | 291 | 299 | OR: 0.97 (0.81–1.16) | Maternal baseline, social class, and other factors |
|  |  |  |  |  | Medication for chest problems in last year | 257 | 285 | OR: 0.88 (0.73–1.06) | Maternal baseline, social class, and other factors |
|  |  |  |  |  | Admission to hospital in last year | 220 | 225 | OR: 0.97 (0.80–1.19) | Maternal baseline, social class, and other factors |
|  |  |  |  |  | Admission for chest problems | 33 | 37 | OR: 0.89 (0.55–1.43) | Maternal baseline, social class, and other factors |
|  |  |  |  |  | Diabetes | 2 | 0 | - | - |
|  |  |  |  |  | All bowel disorders | 54 | 48 | OR: 1.13 (0.76–1.68) | Maternal baseline, social class, and other factors |
|  |  |  |  | Any amoxicillin–clavulanate, 375mg (n=2304), no amoxicillin–clavulanate (n=2350) | Stillbirths | 20 | 24 | OR: 0.85 (0.47–1.54) | Maternal baseline, social class, and other factors |
|  |  |  |  |  | Deaths in first year | 49 | 53 | OR: 0.94 (0.63–1.39) | Maternal baseline, social class, and other factors |
|  |  |  |  |  | Deaths after first year | 6 | 4 | OR: 1.53 (0.43–5.42) | Maternal baseline, social class, and other factors |
|  |  |  |  |  | Total deaths | 75 | 81 | OR: 0.94 (0.68–1.30) | Maternal baseline, social class, and other factors |
|  |  |  |  | Any amoxicillin–clavulanate, 375 mg (n=1608), no amoxicillin–clavulanate (n=1631) | Educational attainment (children failing to achieve level 2 or higher in national curriculum tests) | Reading: 366  Writing: 395  Maths: 230 | Reading: 378  Writing: 431  Maths: 234 | OR: 0.99 (0.95–1.03)  OR: 0.99 (0.95–1.02)  OR: 0.99 (0.95–1.03) | Maternal baseline, social class, and other factors |
| Keski-Nisula 2013^18^  Finland | Prospective cohort | | 45 mother-infant pairs immediately after birth  (17 treated, 28 not treated) | Intrapartum antibiotics according to hospital protocol including GBS, PROM, caesarean section, chorioamnionitis  Intravenous penicillin or amoxicillin in vaginal deliveries and intravenous second-generation cephalosporins in Caesarean deliveries | Lactobacillus-dominant mixed flora transmission | 1 | 13 | OR: 0.08 (0.007–0.80) | Fetal sex, maternal smoking during pregnancy, meconium in amniotic fluid, duration of ruptured membranes |
| Keuchkerian 2005^19^  Uruguay | Randomised controlled trial | | 96 intrapartum women  (47 treated, 49 not treated) | Preterm labour  Amoxicillin 1000 mg sulbactam 500 mg IV every 8 h during the first 48 h and they continued to receive an oral intake of amoxicillin 250 mg sulbactam 250 mg every 8 h for 5 days | Palpitations, flushes, nausea and vomiting | 2 | 0 | - | - |
|  |  |  |  |  | Asymptomatic bacteriuria | 0 | 1 | - | - |
|  |  |  |  |  | Urinary infection | 1 | 0 | - | - |
| Lin 2006^20^  USA | Retrospective cohort | | 1594 infants  (213 treated, 1378 not treated) | GBS prophylaxis  Penicillin | Respiratory distress | 44 | 95 | RR: 2.62 (1.79–3.83) | Mother’s race, mother’s race unknown, age <20 yr, primigravida, fever during labour, caesarean delivery, Medicaid/public assistance and positive prenatal culture for GBS, missing values of rupture of membranes and prenatal cultures the degree of colonisation, gestational age by week, race, insulin requirement during pregnancy, suspected infection during labour, intrauterine catheter, unknown Pitocin use, unknown prenatal GBS culture |
|  |  |  |  |  | Discharge diagnosis of a respiratory disorder | 12 | 39 | *RR: 1.96 (1.04-3.69)* | None |
| Mazzola 2016^21^  Italy | Prospective cohort | | 26 infants  7-30 days old  (13 treated [7 breastfed and 6 mixed fed]  13 not treated [7 breastfed and 6 mixed fed]) | GBS prophylaxis  2g ampicillin at least 4 h before delivery, followed by 1g to maximum 4g. | Gut microbiota composition | At the phylum level, in breastfed infants, at day 7 *Actinobacteria* were not detected in IAP infants and were present at 17% in control infants (p < 0.001) and there were significantly higher abundances of *Proteobacteria* in IAP infants than controls (p < 0.062).  IAP infants were dominated by genera belonging to the *Enterobacteriaceae* family (p = 0.044), particularly *Escherichia*, which accounted for 52% of the total relative abundance, compared with 14% in the control group. *Bifidobacteria* were not detected in any of the IAP infants at day 7 but 16% of the relative abundance from control infants (p = 0.001), and control infants also had higher levels of *Bacteroides* than IAP infants (20% vs. 7%), although not statistically significant (p = 0.078).  At day 30, *Bifidobacteria* numbers appeared to have recovered in the IAP group accounting for 6% of the relative abundance (p = 0.025) in both groups; *Enterobacteriaceae* continued to dominate in IAP infants compared to control infants (44% vs.16%); Additionally, there was a significantly higher level of the *Veillonellaceae* family in control infants compared to IAP infants (p = 0.035).  *Veillonella* is affected by the antibiotic treatment, as it does not increase in the IAP group between 7 and 30 days, whereas a strong increase is shown within control samples at the same sampling times in control samples.  At the phylum level, in mixed-fed infants, at day 7 there was a higher abundance of *Proteobacteria*, (37% vs. 17%) and *Firmicutes* (41% versus 29%) in infant and control infants. On the other hand, *Actinobacteria* (8% vs. 1%) and *Bacteroidetes* (36% vs. 21%) were highest in the control compared to IAP group.  At day 7, IAP infants contained high abundances of organisms belonging to family *Enterobacteriaceae* (35% vs. 17%), and *Streptococcus* (32% vs. 10%) compared to the control group. Control infants had higher levels of *Bacteroides*, (32% vs. 13%), and *Bifidobacterium* (5% vs. 1%), compared with IAP infants.  By day 30, *Actinobacteria* levels increased in the IAP infants to 7% and *Firmicutes* and *Proteobacteria* reduced to 30% and 28%. *Bacteroidetes* were the dominant phylum in both groups, representing 26% in control and 34% in IAP treated infants. At genus level, the microbiota composition was more uniform than that at day 7. Members of the *Enterobacteriaceae* family fall to 28% and *Streptococcus* was significantly reduced to 8% (p = 0.042); The *Lachnospiraceae* family, absent at day 7 in IAP treated infants, was detected at 4% at day 30. *Bifidobacteria* significantly increased in IAP infants from 0% at day 7 to 6% at day 30, (p = 0.013) and remain highest in control infants (19%). | | | None |
|  |  |  |  |  | Gut microbiota sample richness and biodiversity | Alpha diversity  In breastfed infants, at day 7 there was a significantly lower diversity in breastfed IAP infants compared with breast fed control (Chao1 p = 0.012), Simpson p = 0.035, Shannon p = 0.0082 and observed species p = 0.021). By day 30 the Chao index and observed species increased in the IAP infants, although this was not a significant increase, and the Simpson and Shannon indices remained largely unchanged.  In mixed-fed infants, at day 7 there were no significant differences in diversity although Chao1, Shannon and observed species indices were highest in the control infants. At day 30, alpha diversity was similar in both MF groups  Beta diversity  In breastfed infants, at day 7, principal coordinate analysis (PCoA) plots constructed using unweighted UniFrac distance matrices shows clear separation IAP samples from those of the Control infants. By day 30, no clear separation was observed, suggesting that microbial communities became more uniform over time.  In mixed-fed infants, principal coordinate analysis showed no clustering of samples from either IAP or control groups at day 7 or 30. | | | None |
|  |  |  |  |  | Absolute quantification of total bacteria and *bifidobacteria* | Total bacteria numbers were similar across the four groups, ranging between 9.38 to 9.71 at day 7 and 9.53 to 9.83 log CFU/g at day 30, with no significant differences between groups observed  At day 7, *Bifidobacterium* spp numbers were observed to be significantly lower in IAP infants compared with control infants (breast fed IAP vs. control: 5.86 log CFU/g vs. 8.16 log CFU/g, p = 0.005; mixed fed IAP vs. control: 5.81 log CFU/g vs. 7.19 log CFU/g, p = 0.03).  By day 30, a significant increase was observed in both IAP groups (breast fed: 7.72 log CFU/g, p=0.035 and mixed fed: 8.50 log CFU/g, p = 0.036). Numbers remain higher in breast fed control infants compared to IAP infants (8.62 log CFU/g vs. 7.72 log CFU/g) but this no longer a significant difference. Numbers have significantly increased in mixed fed control infants to 8.55 log CFU/g (p = 0.028) and are similar to that of IAP infants, 8.50 log CFU/g. | | | None |
| McGregor 1986^22^  USA | Randomised controlled trial | | 58 intrapartum women  (29 treated, 29 not treated) | Preterm labour  21 enteric-coated erythromycin tablets over 7 days | Withdrawal from study due to nausea/and or vomiting | 1 | 1 | *RR: 1.00 (0.07-15.24)* | - |
| Rajaei 2006^23^  Iran | Randomised controlled trial | | 80 Intrapartum women  (38 treated, 42 not treated) | Preterm labour  400 mg erythromycin every 6 h orally for 10 days. | Side effects: nausea, vomiting, hot flushes, decreased deep tendon reflexes, emotional disturbances or drug intolerance | - | - | No significant difference in side effects | - |
| Roca 2016^24^  Gambia | Randomised controlled trial | | 829 intrapartum women and 843 infants  (414 women and 419 infants treated, 415 women and 424 infants not treated) | Neonatal sepsis prevention  A single dose of oral 2g azithromycin (4 tablets of 0.5g) | Adverse events/serious adverse events in newborns | 0 | - | - | - |
|  |  |  |  |  | Moderate urticarial rash | 1 | - | *-* | - |
|  |  |  |  |  | Newborn GBS nasopharyngeal samples resistant to Azithromycin | Day 2: 0  Day 3: 0  Day 6: 1 (0.3%)  Day 14: 1 (0.3%)  Day 28: 1 (0.3%) | Day 2: 0  Day 3: 2 (0.5%)  Day 6: 2 (0.5%)  Day 14: 1 (0.3)  Day 28: 0 | -  -  PR: 0.51 (0.05–5.62)  PR: 1.02 (0.06–16.31)  - | None |
|  |  |  |  |  | Newborn *S. pneumoniae* nasopharyngeal samples resistant to Azithromycin | Day 2: 0  Day 3: 0  Day 6: 0  Day 14: 2 (0.5%)  Day 28: 8 (2.2%) | Day 2: 0  Day 3: 0  Day 6: 2 (0.5%)  Day 14: 4 (1.0%)  Day 28: 8 (2.1%) | *-*  *-*  *-*  PR: 0.51 (0.009–2.78)  PR: 1.04 (0.40–2.75) | None |
|  |  |  |  |  | Newborn *S. aureus* nasopharyngeal samples resistant to Azithromycin | Day 2: 3 (0.7%)  Day 3: 41 (10.6%)  Day 6: 48 (12.7%)  Day 14: 57 (15.3%)  Day 28: 60 (16.7%) | Day 2: 4 (1.0%)  Day 3: 27 (6.8%)  Day 6: 20 (5.2%)  Day 14: 13 (3.4%)  Day 28: 17 (4.5%) | PR: 0.77 (0.17–3.40)  PR: 1.56 (0.98–2.48)  PR: 2.46 (1.49–4.06)  PR: 4.49 (2.50–8.06)  PR: 3.68 (2.19–6.18) | None |
|  |  |  |  |  | Newborn any bacteria nasopharyngeal samples resistant to Azithromycin | Day 2: 3 (0.7%)  Day 3: 41 (10.6%)  Day 6: 49 (13.0%)  Day 14: 60 (16.1%)  Day 28: 69 (19.2%) | Day 2: 4 (1.0%)  Day 3: 29 (7.3%)  Day 6: 24 (6.2%)  Day 14: 17 (4.5%)  Day 28: 25 (6.7%) | PR: 0.77 (0.17–3.40)  PR: 1.45 (0.92–2.28)  PR: 2.09 (1.31–3.34)  PR: 3.61 (2.15–6.08)  PR: 2.88 (1.86–4.44) | None |
|  |  |  |  |  | Maternal GBS nasopharyngeal samples resistant to Azithromycin | Day 2: 0  Day 3: 0  Day 6: 0  Day 14: 0  Day 28: 1 (0.3%) | Day 2: 0  Day 3: 0  Day 6: 0  Day 14: 0  Day 28: 1 (0.3%) | *-*  *-*  *-*  *-*  PR: 1.01 (0.06–16.1) | None |
|  |  |  |  |  | Maternal *S. pneumoniae* nasopharyngeal samples resistant to Azithromycin | Day 2: 6 (1.4%)  Day 3: 3 (0.8%)  Day 6: 3 (0.8%)  Day 14: 7 (1.8%)  Day 28: 7 (1.8%) | Day 2: 0  Day 3: 2 (0.5%)  Day 6: 4 (1.0%)  Day 14: 3 (0.8%)  Day 28: 1 (0.3%) | *-*  PR: 1.51 (0.25–8.97)  PR: 0.75 (0.17–3.35)  PR: 2.33 (0.61–8.96)  PR: 7.09 (0.88–57.4) | None |
|  |  |  |  |  | Maternal *S. aureus* nasopharyngeal samples resistant to Azithromycin | Day 2: 6 (1.4%)  Day 3: 16 (4.0%)  Day 6: 22 (5.6%)  Day 14: 36 (9.2%)  Day 28: 48 (12.6%) | Day 2: 11 (2.7%)  Day 3: 7 (1.7%)  Day 6: 14 (3.5%)  Day 14: 12 (3.1%)  Day 28: 11 (2.8%) | PR: 0.55 (0.20–1.46)  PR: 2.30 (0.96–8.97)  PR: 1.58 (0.82–3.04)  PR: 3.00 (1.85–5.68)  PR: 4.42 (2.33–8.38) | None |
|  |  |  |  |  | Maternal any bacteria nasopharyngeal samples resistant to Azithromycin | Day 2: 12 (2.9%)  Day 3: 19 (4.8%)  Day 6: 25 (6.3%)  Day 14: 4 (10.5%)  Day 28: 56 (14.7%) | Day 2: 11 (2.7%)  Day 3: 9 (2.2%)  Day 6: 14 (3.5%)  Day 14: 15 (3.8%)  Day 28: 13 (3.4%) | PR: 1.09 (0.49–2.45)  PR: 2.12 (0.97–4.63)  PR: 1.48 (0.81–2.69)  PR: 2.73 (1.54–4.86)  PR: 4.36 (2.43–7.85) | None |
|  |  |  |  |  | Maternal GBS vaginal samples resistant to Azithromycin | Day 2: 1 (0.2%)  Day 8-10: 6 (1.5%) | Day 2: 2 (0.5%)  Day 8-10: 1 (0.3%) | PR: 0.50 (0.05–5.51)  PR: 6.06 (0.73–50.1) | None |
|  |  |  |  |  | Maternal *S. pneumoniae* vaginal samples resistant to Azithromycin | Day 2: 0  Day 8-10: 0 | Day 2: 0  Day 8-10: 0 | -  - | None |
|  |  |  |  |  | Maternal *S. aureus* vaginal samples resistant to Azithromycin | Day 2: 0  Day 8-10: 27 (6.9%) | Day 2: 7 (1.7%)  Day 8-10: 4 (1.0%) | -  PR: 6.82 (2.41–19.3) | None |
|  |  |  |  |  | Maternal any bacteria vaginal samples resistant to Azithromycin | Day 2: 1 (0.2%)  Day 8-10: 32 (8.4%) | Day 2: 9 (2.2%)  Day 8-10: 5 (1.3%) | PR: 0.11 (0.01–0.88)  PR: 6.67 (2.63–16.9) | None |
|  |  |  |  |  | Maternal GBS breast milk samples resistant to Azithromycin | Day 3: 1 (0.3%)  Day 6: 1 (0.3%)  Day 14: 1(0.3%)  Day 28: 0 | Day 3: 2 (0.5%)  Day 6: 2 (0.5%)  Day 14: 1 (0.3%)  Day 28: 0 | PR: 0.51 (0.05–5.56)  PR: 0.50 (0.05–5.52)  PR: 1.00 (0.006–15.9)  - | None |
|  |  |  |  |  | Maternal *S. pneumoniae* breast milk samples resistant to Azithromycin | Day 3: 0  Day 6: 0  Day 14: 0  Day 28: 0 | Day 3: 0  Day 6: 0  Day 14: 0  Day 28: 0 | -  -  -  - | None |
|  |  |  |  |  | Maternal *S. aureus* breast milk samples resistant to Azithromycin | Day 3: 19 (4.8%)  Day 6: 20 (5.1%)  Day 14: 22 (5.7%)  Day 28: 14 (3.7%) | Day 3: 7 (1.8%)  Day 6: 8 (2.0%)  Day 14: 3 (0.8%)  Day 28: 5 (1.3%) | PR: 2.75 (1.17–6.47)  PR: 2.51 (1.12–5.64)  PR: 7.31 (2.21–24.2)  PR: 2.82 (1.03–7.76) | None |
|  |  |  |  |  | Maternal any bacteria breast milk samples resistant to Azithromycin | Day 3: 20 (5.1%)  Day 6: 20 (5.3%)  Day 14: 23 (5.9%)  Day 28: 14 (3.7%) | Day 3: 9 (2.3%)  Day 6: 10 (2.5%)  Day 14: 4 (1.0%)  Day 28: 5 (1.3%) | PR: 2.25 (1.04–4.88)  PR: 2.11 (1.01–4.42)  PR: 5.74 (2.00–16.4)  PR: 2.82 (1.03–7.76) | None |
| Salman 2015^25^  Gambia | Sub-study of Roca et al.’s^24^ randomised controlled trial | | 40 infants from Roca et al.^24^ (20 treated, 20 not treated) | Neonatal sepsis prevention  A single dose of oral 2g azithromycin (4 tablets of 0.5g) | Infantile hypertrophic pyloric  Stenosis (IHPS) | 0 | 0 | - | - |
|  |  |  | 419 newborns from Roca et al.^24^ (all treated) |  |  | 0 | - | 95% CI: 0-11.3/1,000 cases | - |
| Sinha 2003^26^  USA | Case-control study | | 228 infants  0-30 days old  (114 cases of non-GBS infection and 114 controls, 17 infants were treated | GBS prophylaxis  Penicillin G (41%= 7 people), ampicillin (41%= 7 people), clindamycin (18%= 3 people) | Bloodstream infection | - | - | RR: 0.20 (0.011-3.6) | Sex and year of birth |
|  |  |  |  |  | Pneumonia | - | - | RR: 2.5 (0.43-14.0) | Sex and year of birth |
|  |  |  |  |  | Any infection syndrome | - | - | RR: 1.0 (0.38-2.9) | Sex and year of birth |
| Stoll 2002^27^  USA | Retrospective cohort study | | 5447 intrapartum women  (3554 treated, 1893 not treated) | Indication unknown  Ampicillin (49%), penicillin (14%), and erythromycin (13%) | Early-onset sepsis | 63 | 21 | OR: 1.1 (0.6–1.8) | Gestational age, the presence or absence of intrauterine growth restriction, birth weight, race or ethnic group, and sex |
|  |  |  |  |  | *E. coli* Sepsis or death | - | - | No association was found for any maternal antibiotic (data was not shown). | None |
|  |  |  | 33 infants  (28 treated, 5 not treated) | Indication unknown  Ampicillin | Ampicillin-resistant *E. coli* | 26 | 1 | p = 0.01 | None |
|  |  |  | 5447 intrapartum women | Indication unknown  IAP within 72 hours (3399), no IAP within 72 hours (2048)  Indication unknown  Ampicillin IAP within 72 hours (2348), no ampicillin IAP within 72 hours (3099) | Early onset sepsis | 58 | 26 | OR: 1.0 (0.6–1.6) | Gestational age, the presence or absence of intrauterine growth restriction, birth weight, race or ethnic group, and sex |
|  |  |  |  |  | *E. coli* sepsis | 25 | 12 | p = 0.004 | NS  When gestational age and interval between membrane rupture and delivery adjusted for |
| Svare 1997^28^  Denmark | Randomised controlled trial | | 110 intrapartum women  (59 treated, 51 not treated) | Preterm labour  Ampicillin 2g intravenously every six hours for 24 hours followed by  pivampicillin 500 mg orally every eight hours for seven days, plus metronidazole 500 mg intravenously every eight hours for 24 hours followed by metronidazole 400 mg orally every eight hours for seven days | Side effects and allergic reactions (undefined) | 4 | 1 | *RR: 3.46 (0.40-29.95)* | None |
| Wohl 2015^29^  USA | Retrospective cohort study | | 492 children  2 years old  (128 treated, 364 not treated) | Indication unknown  Penicillins (108), macrolides (16), aminoglycosides (3), cephalosporins (1) | Diagnosing atopic dermatitis | Any IAP 37  IAP 0-4 hours: 9/28  IAP 4-12 hours: 11/53  IAP 12-24 hours: 7/26  IAP >24 hours: 6/11 | 100 | RR: 1.03 (0.75–1.41)  RR 1.17 (0.66–2.06)  RR 0.76 (0.44–1.31)  RR 0.98 (0.51–1.89)  RR 1.99 (1.13–3.49) | None |
| **Outcomes used to investigate IAP effectiveness (benefits) in randomised controlled trials** | | | | | | | | | |
| Cox 1996^9^  USA | | Randomised controlled trial | 82 infants  (40 treated, 42 not treated) | Preterm labour  2g ampicillin and 1g sulbactam parenterally every 6 hours for 8 doses, followed by ampicillin-clavunate 250mg orally every 8 hours for 5 days. | 5 minute APGAR score < 7 | 1 | 1 | *RR: 1.05 (0.07-16.23)* | None |
|  |  |  |  |  | Neonatal ICU days | M: 19 (SEM: 0.2, R: 0-21) | M: 22 (SEM: 0.2, R: 0-27) | NS | None |
|  |  |  |  |  | Respiratory distress ventilation | 8 | 8 | NS (unable to calculate RR as some missing) | None |
|  |  |  |  |  | Necrotizing enterocolitis | 0 | 1 | NS (unable to calculate RR as some missing) | None |
|  |  |  |  |  | Still birth | 0 | 0 | NS (unable to calculate RR as some missing) | None |
|  |  |  |  |  | Neonatal death | 1 | 0 | NS (unable to calculate RR as some missing) | None |
| Gordon 1995^12^  USA | | Randomised controlled trial | 117 intrapartum women  (58 treated, 59 not treated) | Preterm labour  Ceftizoxime for 5 days or 3 days | Maternal infection | 2 | 3 | *RR: 0.68 (0.12-3.91)* | None |
|  |  |  |  |  | Neonatal pneumonia | 0 | 0 | NS | None |
|  |  |  |  |  | Neonatal sepsis | 0 | 0 | NS | None |
|  |  |  |  |  | Neonatal positive cultures | 2 | 2 | *RR: 1.02 (0.15-6.98)* | None |
| Kampikaho 1993^14^  Uganda | | Quasi-randomised controlled trial | 660 intrapartum women  (167 streptomycin, 163 penicillin, 330 not treated) | Post-partum infection prevention  1g streptomycin (n=167) or 0.8MU penicillin (n=163) | Laboratory-confirmed post-partum infection | Streptomycin: 14/167  Penicillin: 15/163 | 51/330 | 1.00 (reference)  Streptomycin RR: 0.54 (0.31-0.95)  Penicillin RR: 0.60 (0.35-1.03) | None |
| Keettel 1949^15^  USA | | Randomised controlled trial | 895 intrapartum women (465 treated, 430 not treated) | Post-partum infection prevention  300,000/600,000 units of penicillin at the indication of labour and then after 24-hour intervals. | Puerperium fever | 66 | 89 | *RR: 0.69 (0.51-0 92)* | None |
|  |  |  |  |  | Puerperium Endometritis | 13 | 40 | *RR: 0.30 (0.16-0.55)* | None |
|  |  |  |  |  | Puerperium Pyelitis | 4 | 1 | *RR: 3.70 (0.42-32.96)* | None |
|  |  |  |  |  | Puerperium Mastitis | 5 | 3 | *RR: 1.54 (0.37-6.41)* | None |
|  |  |  |  |  | Stillbirths | 9 | 12 | *RR: 0.69 (0.30-1.63)* | None |
|  |  |  |  |  | Neonatal deaths | 12 | 12 | *RR: 0.92 (0.42-2.04)* | None |
| Keettel 1950^16^  USA | | Controlled trial | 773 intrapartum women  (382 treated, 391 not treated) | Post-partum infection prevention  600,000 units of penicillin at the indication of labour, and then after 24-hour intervals. | Fever | 29 | 61 | *RR: 0.49 (0.32-0.74)* | None |
|  |  |  |  |  | Stillbirth | 5 | 3 | *RR: 1.71 (0.41-7.09)* | None |
|  |  |  |  |  | Neonatal death | 4 | 2 | *RR: 2.05 (0 38-11.11)* | None |
| Keuchkerian 2005^19^  Uruguay | | Randomised controlled trial | 96 intrapartum women  (47 treated, 49 not treated) | Preterm labour  Amoxicillin 1000 mg sulbactam 500 mg IV every 8 h during the first 48 h and they continued to receive an oral intake of amoxicillin 250 mg sulbactam 250 mg every 8 h for 5 days | 1 minute APGAR score < 7 | 3 | 2 | RR: 1.57 (0 27-8.94) | None |
|  |  |  |  |  | Respiratory distress syndrome | 3 | 3 | RR: 1.04 (0.22-4.91) | None |
|  |  |  |  |  | Neonatal sepsis | 0 | 0 | - | - |
|  |  |  |  |  | Fetal death | 1 | 1 | RR: 1.04 (0.07-16.19) | None |
|  |  |  |  |  | Neonatal death | 0 | 0 | - | - |
| McGregor 1986^22^  USA | | Randomised controlled trial | 17 intrapartum women  (8 treated, 9 not treated) | Preterm labour  21 enteric-coated erythromycin tablets over 7 days | Maternal days in hospital | M: 6.1  (SD: 4.7, R: 3-15) | M: 6.3  (SD: 6.2, R: 2-18) | NS | - |
|  |  |  |  |  | Amniotic fluid infection | 0 | 0 | - | - |
|  |  |  |  |  | Maternal febrile morbidity | 0 | 0 | - | - |
|  |  |  |  |  | Initial requirement of neonate intermediate or intensive care nursery | 2 | 3 | *RR: 0.75 (0.16-3.41)* | None |
|  |  |  |  |  | Total days in intermediate or intensive care nursery | 9 | 62 | - | - |
|  |  |  |  |  | Total days in any nursery | M: 3  (SD: 2.1) | M: 9.6  (SD: 13.5) | p = 0.08 | - |
|  |  |  |  |  | Neonates treated with antibiotics | 0 | 1 | - | - |
| Nadiasaukiene  1996^30^  Lithuania | | Randomised controlled trial | 102 mother-infant pairs  (44 treated, 58 not treated) | Preterm labour  2 x 5g ampicillin four hours apart or 1 hour before delivery if labour proceeded quickly | 1 minute APGAR score < 7 | 26 | 40 | *RR: 0.86 (0.63-1.16)* | None |
|  |  |  |  |  | Neonate did not survive first week | 8 | 12 | *RR: 0.88 (0.39-1.96)* | None |
|  |  |  |  |  | Neonatal infection | 4 | 38 | *RR: 0.14 (0.05-0.36)* | None |
|  |  |  |  |  | Histological chorioamnionitis | 6 | 28 | *RR: 0.28 (0.13-0.62)* | None |
|  |  |  |  |  | Puerperal uterine infection | 8 | 26 | *RR: 0.41 (0.20-0.81)* | None |
| Rajaei 2006^23^  Iran | | Randomised controlled trial | 80 Intrapartum women  (38 treated, 42 not treated) | Preterm labour  400 mg erythromycin every 6 h orally for 10 days. | Admission to NICU | 14 | 25 | p<0.05  *RR: 0.62 (0.38-1.01)*  *Risk difference:*  *−22.68% (95% CIs: −44.02- −1.34), and p=0.043* | None |
| Roca 2016^24^  Gambia | | Randomised controlled trial | 829 intrapartum women and 843 infants  (414 women and 419 infants treated, 415 women and 424 infants not treated) | Neonatal sepsis prevention  A single dose of oral 2g azithromycin (4 tablets of 0.5g) | Maternal deaths | 0 | 0 | - | - |
|  |  |  |  |  | Puerperal sepsis | 1 | 2 | *RR: 0.50 (0.05-5.51)* | - |
|  |  |  |  |  | Deaths from neonatal sepsis, meningitis, pneumonia | 3 (all underlying conditions) | 4 (no underlying conditions) | *RR: 0.76 (0.17- 3.37)* | - |
|  |  |  |  |  | Apgar scores at birth | 0: 6  1-6: 8  7-10: 402 | 0: 6  1-6: 5  7-10: 408 | *-* | - |
| Svare 1997^28^  Denmark | | Randomised controlled trial | 110 intrapartum women  (59 treated, 51 not treated) | Preterm labour  Ampicillin 2grams intravenously every six hours for 24 hours followed by  pivampicillin 500 mg orally every eight hours for seven days, plus metronidazole 500 mg intravenously every eight hours for 24 hours followed by metronidazole 400 mg orally every eight hours for seven days | Maternal Chorioamnionitis - endometritis | 3 | 0 | - | None |
|  |  |  |  |  | 5 minute APGAR score < 7 | 5 | 1 | *RR: 4.32 (0.52-35.79)* | None |
|  |  |  |  |  | Admission to neonatal department | 23/58 | 32 | *RR: 0.63 (0.43-0.93)* | None |
|  |  |  |  |  | Days in neonatal department | Median: 15.5  (R: 1-60) | Median: 27  (R: 2-121) | - | - |
|  |  |  |  |  | Oxygen /NCPAP /ventilation | M: 9.7  (SD: 15.7) | M: 10.8  (SD: 17.2) | - | - |
|  |  |  |  |  | Neonatal antibiotic days | M: 5.9  (SD: 2.8) | M: 6.6  (SD: 4.2) | - | - |
|  |  |  |  |  | Meningitis, septicaemia, pneumonia | 6/58 | 11 | *RR: 0.48 (0.19-1.20)* | - |

*E coli: Escherichia coli*, CI: confidence interval, CFU: colony forming units, g: grams, GBS: group B *Streptococcus,* IAP: intrapartum antibiotic prophylaxis, IQR: interquartile range, M: mean, OR: Odds ratio, p: probability level, R: range, RR: relative risk, *S. aureus: Staphylococcus aureus*, S. *pneumoniae: Streptococcus pneumoniae*, SEM: Standard error of mean, NS: not significant, UTI: urinary tract infection

* p=0.037

*Figures in italics have been calculated by authors*

**References**

1. Aloisio I, Mazzola G, Corvaglia LT, Tonti G, Faldella G, Biavati B et al. Influence of intrapartum antibiotic prophylaxis against group B Streptococcus on the early newborn gut composition and evaluation of the anti-Streptococcus activity of Bifidobacterium strains. Appl Microbiol Biotechnol. 2014;98(13):6051-60.

2. Aloisio I, Quagliariello A, De Fanti S, Luiselli D, De Filippo C, Albanese D et al. Evaluation of the effects of intrapartum antibiotic prophylaxis on newborn intestinal microbiota using a sequencing approach targeted to multi hypervariable 16S rDNA regions. Appl Microbiol Biotechnol. 2016;100(12):5537-46. doi:<http://dx.doi.org/10.1007/s00253-016-7410-2>.

3. Arboleya S, Sanchez B, Milani C, Duranti S, Solis G, Fernandez N et al. Intestinal Microbiota Development in Preterm Neonates and Effect of Perinatal Antibiotics. J Pediatr. 2015;166(3):538-44.

4. Arboleya S, Sanchez B, Solis G, Fernandez N, Suarez M, Hernandez-Barranco AM et al. Impact of Prematurity and Perinatal Antibiotics on the Developing Intestinal Microbiota: A Functional Inference Study. Int. 2016;17(5). doi:<http://dx.doi.org/10.3390/ijms17050649>.

5. Ashkenazi-Hoffnung L, Melamed N, Ben-Haroush A, Livni G, Amir J, Bilavsky E. The Association of Intrapartum Antibiotic Exposure With the Incidence and Antibiotic Resistance of Infantile Late-Onset Serious Bacterial Infections. Clin Pediatr. 2011;50(9):827-33.

6. Balter S, Zell ER, O'Brien KL, Roome A, Noga H, Thayu M et al. Impact of intrapartum antibiotics on the care and evaluation of the neonate. The Pediatric infectious disease journal. 2003;22(10):853-7. doi:10.1097/01.inf.0000090920.22425.dc.

7. Briody VA, Albright CM, Has P, Hughes BL. Use of Cefazolin for Group B Streptococci Prophylaxis in Women Reporting a Penicillin Allergy Without Anaphylaxis. Obstetrics and gynecology. 2016;127(3):577-83.

8. Corvaglia L, Tonti G, Martini S, Aceti A, Mazzola G, Aloisio I et al. Influence of Intrapartum Antibiotic Prophylaxis for Group B Streptococcus on Gut Microbiota in the First Month of Life. J Pediatr Gastroenterol Nutr. 2016;62(2):304-8.

9. Cox SM, Bohman VR, Sherman ML, Leveno KJ. Randomized investigation of antimicrobials for the prevention of preterm birth. American journal of obstetrics and gynecology. 1996;174(1 Pt 1):206-10.

10. Dinsmoor MJ, Viloria R, Lief L, Elder S. Use of intrapartum antibiotics and the incidence of postnatal maternal and neonatal yeast infections. Obstetrics & Gynecology. 2005;106(1):19-22.

11. Glasgow TS, Young PC, Wallin J, Kwok C, Stoddard G, Firth S et al. Association of intrapartum antibiotic exposure and late-onset serious bacterial infections in infants. Pediatrics. 2005;116(3):696-702.

12. Gordon M, Samuels P, Shubert P, Johnson F, Gebauer C, Iams J. A randomized, prospective study of adjunctive ceftizoxime in preterm labor. American journal of obstetrics and gynecology. 1995;172(5):1546-52.

13. Jaureguy F, Carton M, Panel P, Foucaud P, Butel MJ, Doucet-Populaire F. Effects of intrapartum penicillin prophylaxis on intestinal bacterial colonization in infants. J Clin Microbiol. 2004;42(11):5184-8.

14. Kampikaho A, Irwig LM. A randomized trial of penicillin and streptomycin in the prevention of post-partum infection in Uganda. Int J Gynaecol Obstet. 1993;41(1):43-52.

15. Keettel WC, Scott JW, Plass ED. An evaluation of prophylactic penicillin administration to parturient women. American Journal of Obstetrics & Gynecology. 1949;58(2):335-44. doi:10.1016/0002-9378(49)90387-7.

16. Keettel WC, Plass ED. Prophylactic administration of penicillin to obstetric patients: Additional data. Journal of the American Medical Association. 1950;142(5):324-8. doi:10.1001/jama.1950.02910230026007.

17. Kenyon S, Pike K, Jones DR, Brocklehurst P, Marlow N, Salt A et al. Childhood outcomes after prescription of antibiotics to pregnant women with spontaneous preterm labour: 7-year follow-up of the ORACLE II trial. Lancet. 2008;372(9646):1319-27. doi:<http://dx.doi.org/10.1016/S0140-6736(08)61203-9>.

18. Keski-Nisula L, Kyynarainen HR, Karkkainen U, Karhukorpi J, Heinonen S, Pekkanen J. Maternal intrapartum antibiotics and decreased vertical transmission of Lactobacillus to neonates during birth. Acta Paediatr. 2013;102(5):480-5.

19. Keuchkerian SE, Sosa CG, Fernandez A, Alonso JG, Laborde A, Cuadro JC. Effect of amoxicillin sulbactam in threatened preterm labour with intact membranes: a randomised controlled trial. European journal of obstetrics, gynecology, and reproductive biology. 2005;119(1):21-6. doi:10.1016/j.ejogrb.2004.05.010.

20. Lin FY, Troendle JF. Hypothesis: Neonatal respiratory distress may be related to asymptomatic colonization with group B streptococci. Pediatr Infect Dis J. 2006;25(10):884-8.

21. Mazzola G, Murphy K, Ross RP, Di Gioia D, Biavati B, Corvaglia LT et al. Early gut microbiota perturbations following intrapartum antibiotic prophylaxis to prevent group B streptococcal disease. PLoS ONE. 2016;11 (6) (no pagination)(e0157527). doi:<http://dx.doi.org/10.1371/journal.pone.0157527>.

22. McGregor JA, French JI, Reller LB, Todd JK, Makowski EL. Adjunctive erythromycin treatment for idiopathic preterm labor: results of a randomized, double-blinded, placebo-controlled trial. American journal of obstetrics and gynecology. 1986;154(1):98-103.

23. Rajaei M, Sultani M, Zare S. A randomized controlled trial of adjunctive erythromycin in women with idiopathic preterm labor. The journal of maternal-fetal & neonatal medicine : the official journal of the European Association of Perinatal Medicine, the Federation of Asia and Oceania Perinatal Societies, the International Society of Perinatal Obstet. 2006;19(1):17-20. doi:10.1080/14767050500361455.

24. Roca A, Oluwalana C, Bojang A, Camara B, Kampmann B, Bailey R et al. Oral azithromycin given during labour decreases bacterial carriage in the mothers and their offspring: a double-blind randomized trial. Clin Microbiol Infect. 2016;22(6). doi:10.1016/j.cmi.2016.03.005.

25. Salman S, Davis TM, Page-Sharp M, Camara B, Oluwalana C, Bojang A et al. Pharmacokinetics of Transfer of Azithromycin into the Breast Milk of African Mothers. Antimicrobial agents and chemotherapy. 2015;60(3):1592-9. doi:10.1128/aac.02668-15.

26. Sinha A, Yokoe D, Platt R. Intrapartum antibiotics and neonatal invasive infections caused by organisms other than group B streptococcus. The Journal of pediatrics. 2003;142(5):492-7. doi:10.1067/mpd.2003.154.

27. Stoll BJ, Hansen N, Fanaroff AA, Wright LL, Carlo WA, Ehrenkranz RA et al. Changes in pathogens causing early-onset sepsis in very-low-birth-weight infants. New England Journal of Medicine. 2002;347(4):240-7. doi:<http://dx.doi.org/10.1056/NEJMoa012657>.

28. Svare J, Langhoff-Roos J, Andersen LF, Kryger-Baggesen N, Borch-Christensen H, Heisterberg L et al. Ampicillin-metronidazole treatment in idiopathic preterm labour: a randomised controlled multicentre trial. British journal of obstetrics and gynaecology. 1997;104(8):892-7.

29. Wohl DL, Curry WJ, Mauger D, Miller J, Tyrie K. Intrapartum antibiotics and childhood atopic dermatitis. J Am Board Fam Med. 2015;28(1):82-9. doi:<http://dx.doi.org/10.3122/jabfm.2015.01.140017>.

30. Nadisauskiene R, Bergstrom S. Impact of intrapartum intravenous ampicillin on pregnancy outcome in women with preterm labor: A randomised, placebo-controlled study. Gynecologic and Obstetric Investigation. 1996;41(2):85-8.
